# The molecular mechanisms of Guizhi Fuling Pills in ameliorating Alzheimer’s disease-like cognitive impairment: insights from transcriptomics, metabolomics, and gut microbiome

**DOI:** 10.3389/fnagi.2026.1839445

**Published:** 2026-08-13

**Authors:** Lan Ma, Jing Wang, Zhenghao Xu, Zuxiu Huang, Kelong Chen, Xiaoqiong Teng, Miao Chen, Shuyang Lin, Rong Zhou

**Affiliations:** 1 Wenzhou TCM Hospital of Zhejiang Chinese Medical University, Wenzhou, China; 2 Nanning Hospital of Traditional Chinese Medicine, Nanning, China; 3 Zhejiang Chinese Medical University, Hangzhou, China

**Keywords:** Alzheimer’s disease, Guizhi Fuling Pills, gut microbiota, metabolomics, transcriptomics

## Abstract

**Background:**

Alzheimer’s disease (AD)-like cognitive impairment, as a major type of cognitive disorder, has witnessed a sharp rise in prevalence. Therefore, there is an urgent need to develop effective therapeutic intervention measures. Guizhi Fuling Pills (GFP), a classical Traditional Chinese Medicine (TCM) formula, has been shown to exert protective effects on cognitive function. However, its underlying mechanisms remain unclear.

**Objective:**

To investigate the effects of GFP on AD-like cognitive impairment and elucidate its underlying mechanisms.

**Methods:**

D-galactose (D-gal)-induced aged mice were used as the model. Mice were administered via gavage for 4 weeks with 0.9% normal saline (0.1 mL/10 g/d), low-dose GFP (12.56 g/kg/d), medium-dose GFP (25.11 g/kg/d), high-dose GFP (50.22 g/kg/d), and donepezil (5 mg/kg/d). A behavioral test was conducted using the Morris water maze. Histopathological changes were observed via H&E staining and immunohistochemistry (IHC). In addition, various methods such as transcriptomics, metabolomics, network pharmacology, and analysis of gut microbiota were utilized to elucidate the possible mechanisms.

**Results:**

Guizhi Fuling Pills improved learning and memory function in aged mice, ameliorated hippocampal neuronal morphology, and reduced p-Tau protein deposition. Network pharmacology and hippocampal transcriptomic analyses suggested that the active components in GFP may ameliorate cognitive impairment through multiple mechanisms. It included regulation of the VEGF and PI3K/AKT signaling pathways, attenuation of inflammatory responses, inhibition of apoptosis, and repair of the blood-brain barrier (BBB). Gut microbiota analysis revealed that GFP modulated the compositional structure of the gut microbiota, including increasing the abundance of Lactobacillales and decreasing Desulfovibrionia and Tannerellaceae. Metabolomics suggested that GFP may ameliorate metabolic disorders in aged mice by modulating the synthesis of lipids and lipid-like molecules.

**Conclusion:**

The findings of this study suggest that GFP may ameliorate cognitive dysfunction in AD-like cognitive impairment mice through multiple mechanisms, including repair of the BBB, attenuation of inflammatory responses, and modulation of the gut microbiota and metabolic disorders.

## Graphical Abstract

**Figure:**
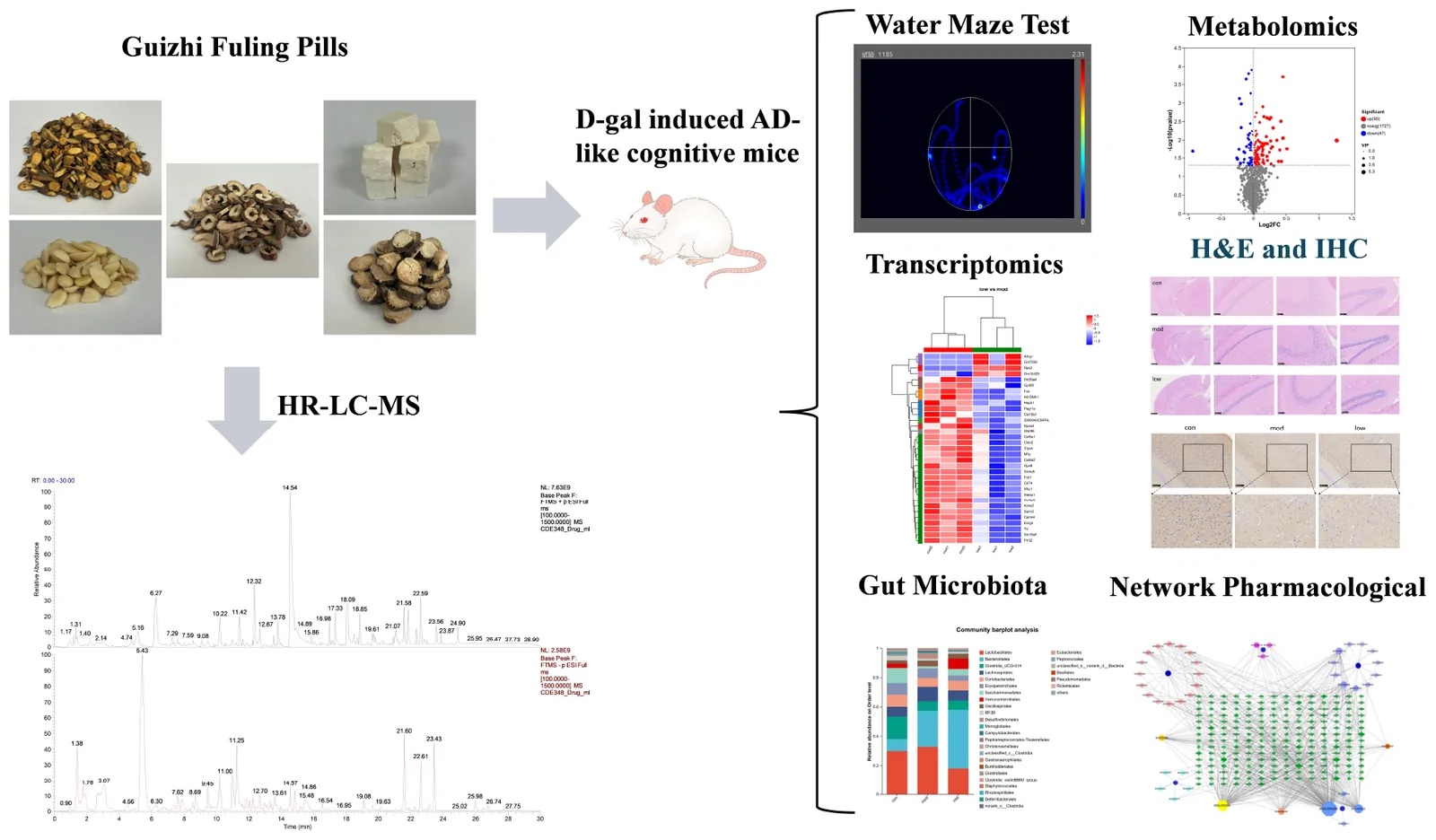
The experimental workflow for GFP. This graphical abstract illustrates the molecular mechanisms underlying the ameliorative effects of Guizhi Fuling Pills on Alzheimer’s disease-like cognitive impairment, based on integrative analyses of transcriptomics, metabolomics, and the gut microbiome.

## 1 Background

With the rapid aging of the global population, the prevalence of cognitive impairment is rising sharply. In 2021, 57 million people were living with dementia worldwide. Every year, there are nearly 10 million new cases (World Health Organization, 2017). The predominant symptoms of cognitive impairment include a decline in learning, memory, information processing, and executive function. Cognitive impairment significantly increases the risk of cardiovascular disease, stroke, and other disorders characterized by insidious onset and progressive cognitive impairment, and represents the leading cause of cognitive impairment, accounting for 60%–80% of all cases (Alzheimers Dement, 2024). It has become a major medical and social challenge, underscoring the urgent need for effective therapeutic interventions for AD-like cognitive impairment. Aging is the most well-established risk factor for AD, and epidemiological data indicate that the incidence of AD increases substantially with age (Alzheimers Dement, 2024). Consequently, the establishment of aged animal models has become an important experimental approach for screening anti-AD drugs and investigating the mechanisms underlying age-related cognitive impairment, as these models can partially recapitulate the high-risk factors for AD pathogenesis. Nevertheless, it must be acknowledged that aged models primarily recapitulate oxidative stress, metabolic disturbances, and physiological decline, and are not yet capable of fully mimicking the core characteristic pathological hallmarks of AD.

Bioactive compounds from TCM have been demonstrated to exert diverse therapeutic effects. For instance, a pectin-like polysaccharide fraction isolated from the rhizome of *Gastrodia elata* exhibits potent anti-inflammatory activity (Akhtar et al., 2025), while jujube (*Ziziphus jujuba*) fermented into vinegar shows potential for ameliorating type 2 diabetes mellitus and cardiovascular diseases (Ali et al., 2025). Accumulating evidence indicates that various active ingredients capable of alleviating cognitive impairment are present in TCM and medicine-food homologous substances (Nie et al., 2024; Rafique et al., 2023). TCM offers the advantages of multi-target effects and relatively fewer adverse reactions; however, due to its complex composition, the underlying mechanisms remain difficult to elucidate, necessitating the integration of multi-omics and network pharmacology approaches to clarify its mechanisms from multiple perspectives. Integrative multi-omics analysis, as a mainstream approach in current systems biology, has been widely applied in drug discovery and development (Dong et al., 2023, 2024; Rafique et al., 2025).

Guizhi Fuling Pills, documented in the ancient Chinese medical classic Synopsis of the Golden Chamber, is composed of five Chinese medicinal herbs: Cinnamomi Ramulus, Poria, Persicae Semen, Paeoniae Radix Rubra, and Moutan Cortex. In recent years, studies have shown that this formula is not only applicable for treating gynecological disorders but has also been clinically observed to exert potential beneficial effects on cognitive function (Di et al., 2021; Yanjie et al., 2019). GFP may confer neuroprotective effects by attenuating inflammatory responses and inhibiting oxidative stress. Its key component, quercetin, alleviates neuroinflammation by downregulating pro-inflammatory cytokines and inhibiting microglial proliferation (Bayazid and Lim, 2022; Gonzales et al., 2022; Le et al., 2020). Furthermore, GFP has been reported to alleviate lipid metabolism disorders and ameliorate vascular endothelial injury (Kou et al., 2022). Although GFP has demonstrated certain therapeutic efficacy against cognitive impairment in clinical settings, its precise mechanisms remain unclear, and its impact on gut microbiota, serum metabolites, and related pathways in AD-like cognitive impairment warrants in-depth investigation. Therefore, we hypothesized that GFP may exert beneficial effects against AD-like cognitive impairment. In the present study, using a D-gal-induced aged model and integrating transcriptomics, metabolomics, and gut microbiota analysis, we aimed to systematically elucidate whether GFP improves AD-like cognitive impairment and to explore its potential mechanisms, thereby providing a promising therapeutic strategy for AD-like cognitive impairment.

## 2 Materials and methods

### 2.1 Drug source, animal modeling, grouping, and intervention

Guizhi Fuling Pills was purchased from Jiangsu Kanion Pharmaceutical Co., Ltd., (National Drug Approval No. Z10950005). All animal procedures were conducted in accordance with the National Institutes of Health Guide for the Care and Use of Laboratory Animals and approved by the Animal Ethics Committee of the Laboratory Animal Center, Wenzhou Institute, University of Chinese Academy of Sciences (Approval No. WIUCAS25031906). KM mice (male, 3 months old, 25–30 g) were obtained from Beijing Vital River Laboratory Animal Technology Co., Ltd. The mice were housed under controlled conditions with a relative humidity of 50 ± 10%, a 12/12 h light–dark cycle, and an ambient temperature of 22 ± 2°C, with free access to standard chow and water.

After 1 week of acclimatization, mice were stratified by body weight and randomly assigned to six groups (*n* = 15 per group) using a random number table: control group, model group, GFP-low group, GFP-medium group, GFP-high group and donepezil group. Except for the control group, all mice were subcutaneously injected with D-gal (150 mg/kg/d) for 8 consecutive weeks, while the control group received an equivalent volume of normal saline.

Starting from the 6th week of modeling, the corresponding treatments were administered by gavage for 4 consecutive weeks. GFP and donepezil were dissolved in normal saline. The dosage of GFP was calculated based on the body surface area conversion formula for animal-to-human equivalence. The GFP-medium dose was set at 25.11 g/kg (equivalent to the clinical daily dose for humans); the GFP-low and GFP-high doses were set at 0.5-fold (12.56 g/kg) and 2-fold (50.22 g/kg) of the medium dose, respectively. Donepezil was administered at 5 mg/kg/d. The control and model groups received an equivalent volume of normal saline (0.1 mL/10 g/d) by gavage. All groups were treated twice daily for 4 weeks.

During the modeling period, one mouse in the GFP-low group died accidentally due to an injection error. Allocation concealment was not implemented for the modelers in this study. Due to the requirements of behavioral testing, the operators were not blinded. Data collection was automatically performed by a video tracking system, and behavioral data were analyzed by personnel who were unaware of the group assignments to ensure objective results. The exclusion criteria for this study were as follows: animals exhibiting abnormal behavior during the Morris water maze training period due to motor impairment or visual deficits, and those with unqualified specimens resulting from operational errors during tissue collection.

### 2.2 Identification of drug constituents

The chemical components of GFP were analyzed and identified using liquid chromatography–mass spectrometry (LC–MS). Briefly, 0.2 g of the herbal powder was accurately weighed and mixed with 1.0 mL of 80% methanol and grinding beads. The mixture was ground for 5 min, vortexed for 10 min, and centrifuged at 13,000 rpm for 10 min. The supernatant was then collected for further analysis.

Mass spectrometry was performed using an electrospray ionization (ESI) source operating in both positive and negative ion switching modes. The acquisition parameters were as follows: full mass/dd-MS^2^ scan mode; resolution, 70,000 for full mass and 17,500 for dd-MS^2^; scan range, 100.0–1500.0 m/z; spray voltage, 3.2 kV (both positive and negative); capillary temperature, 300 °C; collision gas, high-purity argon (purity ≥ 99.999%); normalized collision energies (NCE), 30, 40, and 60; sheath gas, nitrogen (purity ≥ 99.999%) at 40 Arb; auxiliary gas, nitrogen (purity ≥ 99.999%) at 15 Arb and 350 °C. The data acquisition time was 30.0 min. Chromatographic separation was carried out on an AQ-C18 column (150 × 2.1 mm, 1.8 μm, Welch) at a flow rate of 0.30 mL/min. The mobile phase consisted of 0.1% formic acid in water (aqueous phase) and methanol (organic phase). The column oven temperature was set at 35 °C, the autosampler temperature at 10.0 °C, and the injection volume at 5.00 μL.

The data were preliminarily processed using Compound Discoverer 3.3 (CD 3.3, Thermo Fisher Scientific) and subsequently compared against the mzCloud and mzVault databases for compound identification.

### 2.3 Morris water maze

The Morris water maze consisted of a circular pool (diameter: 120 cm; height: 55 cm) divided into four quadrants. A hidden platform was placed in the third quadrant, submerged 1 cm below the water surface. During the first 3 days, the place navigation test was conducted as the learning phase. Each mouse was first placed on the platform for 10 s to observe the surrounding cues and memorize the platform location. Subsequently, the mouse was gently released into the water from the designated navigation markers of the first, second, and fourth quadrants, facing the pool wall. The escape latency required for each mouse to find the hidden platform was recorded. On days 4 and 5, the place navigation test was performed as the testing phase, during which the mice were no longer guided to learn or remember the platform position. On day 6, the spatial probe test was conducted, during which the platform was removed. Each mouse was placed into the water from the quadrant opposite to the original platform quadrant, facing the pool wall. The number of platform crossings within a 60 s swimming period was recorded. All behavioral data were recorded using the EthoVision XT video tracking system.

### 2.4 Transcriptomics

Three mice were randomly selected from each of the model group, GFP-low group, GFP-medium group, and GFP-high group, respectively. Bilateral hippocampal tissues were isolated and preserved at −80°C. Total RNA of hippocampal tissues was extracted using the MJZol total RNA extraction kit (Majorbio Bio-Pharm Technology Co., Ltd., Shanghai, China), and RNA quantification was performed with a NanoDrop 2000 spectrophotometer (Thermo Fisher Scientific, Waltham, MA, USA). Eukaryotic mRNA sequencing was conducted on the NovaSeq X Plus platform, and library construction was implemented following the Illumina NovaSeq Reagent Kit protocol for standard input RNA. Briefly, mRNA containing polyA tails was enriched from total RNA via A-T base pairing with oligo(dT)-coated magnetic beads. Fragmentation buffer was added to randomly shear mRNA into short fragments of approximately 300 bp. First-strand and second-strand cDNA were synthesized through reverse transcription, followed by adapter ligation, fragment screening, and library enrichment. Qualified libraries were sequenced on the NovaSeq X Plus platform (Illumina, USA).

Raw sequencing reads were quality-controlled by fastp^1^. Clean reads after quality filtering were mapped to the reference genome using HISAT2^2^ to generate mapped reads for subsequent transcript assembly and gene expression quantification, with overall mapping quality evaluated simultaneously. Gene and transcript expression abundances were quantified by RSEM^3^. Differential expression analysis between groups was carried out using DESeq2^4^. Genes satisfying the thresholds of FDR < 0.05 and | log2FC | ≥ 1 were defined as differentially expressed genes (DEGs). GO functional enrichment analysis was performed using Goatools^5^ and KEGG pathway enrichment analysis was implemented via KOBAS^6^. Terms and pathways with a corrected *P*-value ≤ 0.05 were considered significantly enriched.

### 2.5 Gut microbiota analysis

Six mice were randomly selected from the control group, model group, and GFP-medium group, respectively. At least three fresh fecal pellets were collected from the rectum of each mouse, transferred into sterile cryogenic tubes, and stored at −80°C until further analysis. Total community genomic DNA was extracted from fecal samples using the FastPure Stool DNA Isolation Kit (MJYH, Shanghai, China) in accordance with the manufacturer’s instructions. The concentration and purity of extracted DNA were determined using a NanoDrop 2000 spectrophotometer (Thermo Fisher Scientific, Waltham, MA, USA). PCR amplification was performed on an ABI GeneAmp^®^ 9700 thermal cycler. Purified PCR amplicons were used to construct sequencing libraries with the NEXTFLEX Rapid DNA-Seq Kit (Bioo Scientific, USA), followed by high-throughput amplicon sequencing. All bioinformatic analyses were completed on the Majorbio Cloud Platform^7^.

Raw paired-end reads were subjected to quality filtering with fastp^8^ (version 0.23.4) and merged by FLASH^9^ (version 1.2.11). The processed sequences were clustered into operational taxonomic units (OTUs) at 97% sequence similarity using USEARCH software^10^ (version 11), with chimeric sequences removed during clustering. Taxonomic annotation of representative OTU sequences was accomplished using the RDP Classifier^11^ (version 2.11) against the Silva v138.2 16S rRNA bacterial database with a confidence cutoff of 70%.

Alpha diversity indices and beta diversity metrics were calculated using Mothur (version 1.30.2), and all visualizations were realized in R software (version 3.3.1). Principal coordinate analysis (PCoA) based on the Bray-Curtis distance matrix combined with ANOSIM/Adonis tests was applied to assess the overall dissimilarity of bacterial communities across sample groups. Kruskal-Wallis test (for multi-group comparisons) and Wilcoxon rank-sum test (for two-group comparisons) were adopted to evaluate intergroup statistical significance. Linear discriminant analysis effect size (LEfSe) analysis (LDA score > 2, *P* < 0.05) was utilized to identify bacterial taxa with significantly differential abundances from phylum to genus levels. Bar charts and heatmaps of community composition were generated to intuitively visualize the distribution of dominant bacterial taxa (Dong et al., 2023).

### 2.6 Untargeted metabolomic analysis

Untargeted metabolomics detection was performed on a UHPLC-Q Exactive HF-X mass spectrometer (Thermo Fisher Scientific, USA) equipped with an ACQUITY HSS T3 column (100 mm × 2.1 mm i.d., 1.8 μm; Waters, Milford, USA). All raw LC-MS data were imported into Progenesis QI (Waters Corporation, USA) for subsequent data processing. (1) Sample Preparation and Metabolite Extraction Six mice were randomly selected from the normal, model, and GFP-medium groups, respectively. Serum samples were thawed on ice after retrieval from -80 °C storage. Briefly, 100 μL of serum was mixed with 300 μL of extraction solution (acetonitrile: methanol = 1:1, v/v), vortexed for 30 s, and subjected to low-temperature ultrasonic extraction for 30 min (5°C, 40 kHz). The mixture was incubated at -20 °C for 30 min to precipitate proteins, then centrifuged at 13,000 *g* for 15 min at 4 °C. The supernatant was transferred and dried under nitrogen flow, reconstituted in 100 μL of reconstitution solution, followed by low-temperature sonication for 5 min. After centrifugation at 13,000 *g* for 10 min at 4 °C, the supernatant was collected for LC-MS/MS analysis. Equal volumes of supernatant from all individual samples were pooled to prepare quality control (QC) samples. All LC-MS detection was completed by Majorbio Bio-Pharm Technology Co., Ltd., (Shanghai, China). (2) LC-MS/MS Detection Conditions Metabolite separation and mass spectrometry acquisition were carried out on a UHPLC-Exploris 240 system (Thermo Fisher Scientific, USA) coupled with an ACQUITY HSS T3 chromatographic column (100 mm × 2.1 mm i.d., 1.8 μm; Waters, Milford, USA). Mobile phase A consisted of a water–acetonitrile mixture (95:5, v/v) containing 0.1% formic acid, and mobile phase B was a ternary mixture of acetonitrile: isopropanol: water (47.5:47.5:5, v/v/v) supplemented with 0.1% formic acid. The injection volume was 3 μL, and the column temperature was maintained at 40 °C. Electrospray ionization (ESI) was operated in both positive and negative ion modes for mass spectrum acquisition. The mass scan range was set at m/z 70–1050. Key MS parameters were set as follows: sheath gas flow rate, 60 arb; auxiliary gas flow rate, 20 arb; ion source heater temperature, 350°C; spray voltage, 3400 V (positive mode) and -3000 V (negative mode); normalized collision energies, 20%, 40%, and 60%. QC samples were prepared by mixing equal volumes of extracts from all biological samples, processed and tested identically to experimental samples. One QC sample was inserted every five analytical injections throughout the whole detection process to monitor instrumental stability and reproducibility. (3) Data Processing and Statistical Analysis Raw LC-MS data were imported into Progenesis QI v3.0 (Waters Corporation, Milford, USA) for baseline filtering, peak identification, peak integration, retention time alignment, and peak matching, generating a data matrix containing retention time, mass-to-charge ratio (m/z), and peak intensity information. Feature annotation was performed within the same software, with MS mass error strictly limited to <10 ppm. Metabolite identification was validated by matching MS/MS fragmentation spectra against public databases including HMDB^12^, Metlin^13^, and the in-house metabolite database of Majorbio. The R package “ropls” was used to perform principal component analysis (PCA) and orthogonal partial least squares discriminant analysis (OPLS-DA) on the preprocessed data matrix. Metabolites with variable importance in projection (VIP) > 1 and *P* < 0.05 were screened as significantly differential metabolites based on OPLS-DA VIP values and univariate statistical tests. Differential metabolites were mapped to biological pathways via the KEGG database, and pathway enrichment analysis was implemented using the Python package “scipy.stats” with Fisher’s exact test to identify pathways significantly perturbed by experimental interventions.

### 2.7 Network pharmacological analysis

Active ingredients and corresponding targets of the five constituent herbs of GFP (i.e., Cinnamomi Ramulus, Poria, Persicae Semen, Paeoniae Radix Rubra, and Moutan Cortex) were retrieved from the TCMSP database. AD-related targets were collected from DrugBank, OMIM, GeneCards, DisGeNET, and TTD databases. Intersection targets between drug-related and disease-related targets were identified using Venn diagram analysis. Protein–protein interaction (PPI) analysis was performed using the STRING database, and the PPI network was visualized using Cytoscape software (version 3.7.2). The potential targets were imported into the DAVID database for Gene Ontology (GO) functional annotation and Kyoto Encyclopedia of Genes and Genomes (KEGG) pathway enrichment analysis. Related network relationships were visualized using Cytoscape 3.7.2.

### 2.8 Hematoxylin and eosin (H&E) staining

After the behavioral tests, the animals were anesthetized and transcardially perfused with PBS, followed by perfusion with 4% paraformaldehyde solution for fixation. The brain tissues were collected and post-fixed in 4% paraformaldehyde at 4 °C for 24 h, and then subjected to gradient dehydration, clearing, and paraffin embedding. Sections of 4 μm thickness were cut using a paraffin microtome (RM2235, Leica) and stained with H&E. All sections were captured and imaged using a microscope slide scanner (DM3000, Leica).

### 2.9 Immunohistochemistry (IHC)

After fixation in 4% paraformaldehyde for 24 h, the tissues were embedded in paraffin, sectioned, and dewaxed. The sections were permeabilized with blocking/permeabilization solution for 30 min at room temperature in the dark, and then subjected to antigen retrieval in 0.01 M sodium citrate buffer (pH 6.0) using high-power heating in a microwave oven. After blocking of non-specific proteins, the sections were incubated with primary antibody against p-Tau (Ser396) (Biodragon, RM7436) at a dilution of 1:500 overnight at 4 °C. Subsequently, the sections were incubated with biotinylated secondary antibody (biotinized sheep anti-rabbit) in a 37°C constant-temperature oven for 30 min, followed by incubation with SP (streptavidin-peroxidase) at 37°C for 30 min. DAB (3,3’-diaminobenzidine) was then added, and the staining reaction was monitored and terminated after approximately 3 min by discarding the chromogen solution. The sections were then dehydrated with absolute ethanol, cleared, and sealed with neutral resin. Images were captured using a light microscope (DM3000, Leica).

### 2.10 Statistic

All data are presented as mean ± standard error of the mean (SEM). Statistical analyses were performed using GraphPad Prism 8.0.1 (GraphPad Software, San Diego, CA, USA). The mean escape latency in the Morris water maze test was compared among groups using a mixed-effects model ANOVA, while other comparisons among groups were analyzed by one-way analysis of variance (ANOVA). A value of *P* < 0.05 was considered statistically significant.

## 3 Results

### 3.1 Identification results of GFP

**FIGURE 1:**
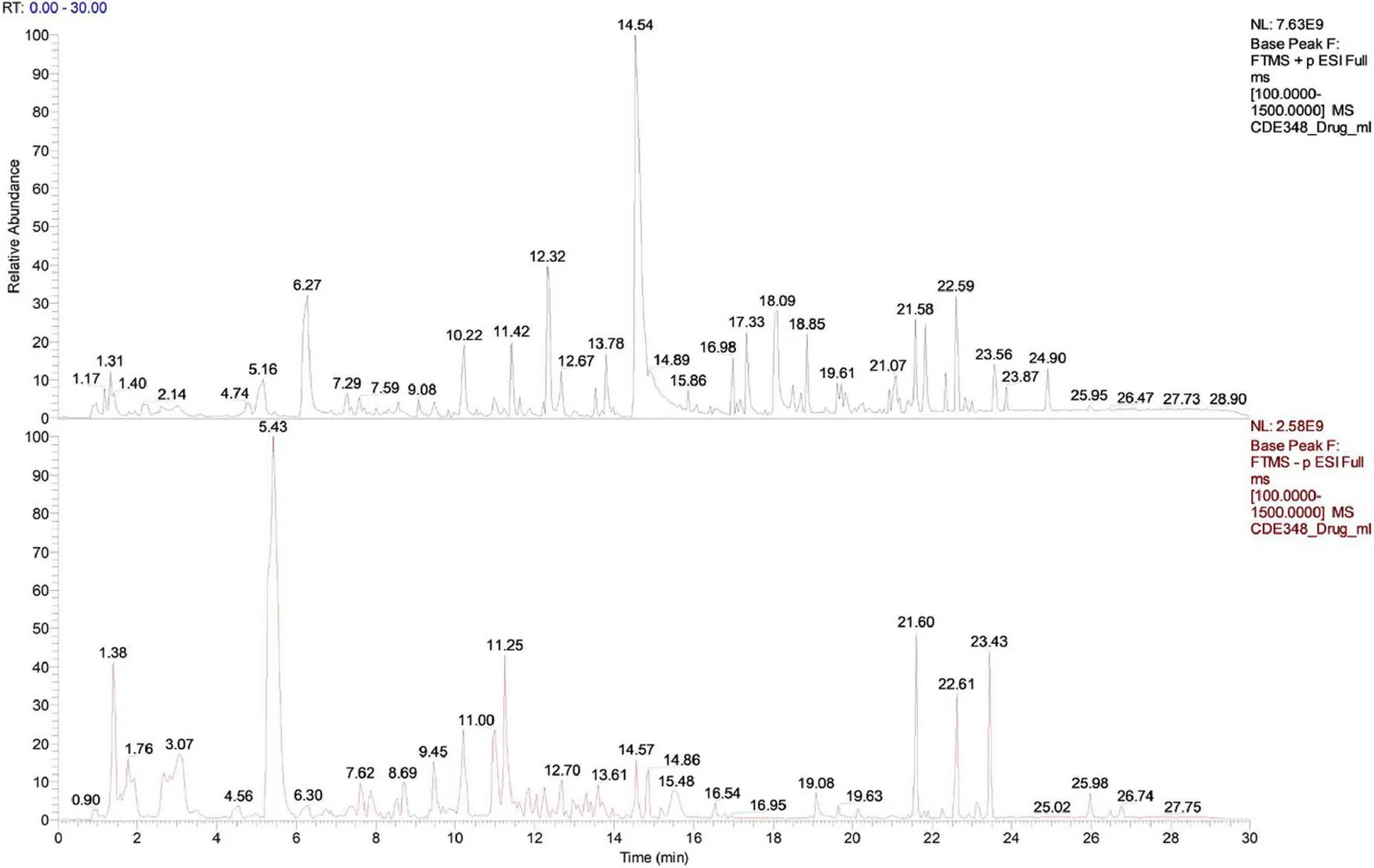
Total Ion Chromatogram (TIC) of chemical composition identification.

A total of 473 compounds were matched in the databases, with the main ones including o-veratraldehyde, cinnamaldehyde, albiflorin, and paeoniflorin. Among these, 126 compounds exhibited an absolute Annot. DeltaMass (ppm) of less than 5, and 87 compounds had an mzVault Best Match score greater than 70 (see Supplementary Table 1 for details). The total ion chromatogram (TIC) of the chemical components is shown in Figure 1.

### 3.2 Morris water maze

**FIGURE 2:**
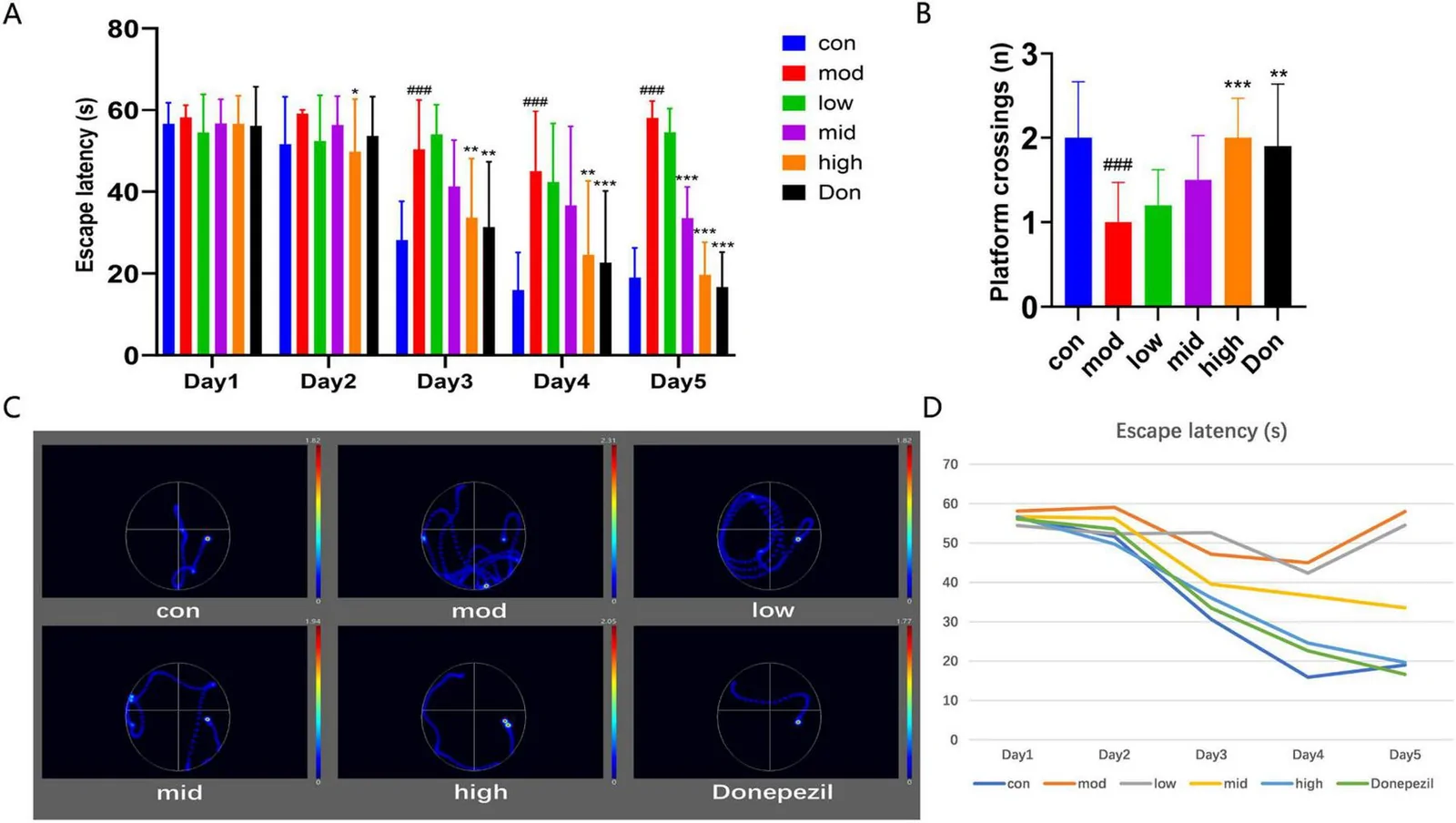
Morris water maze analysis. **(A)** Mean escape latency of mice in each group during days 1–5. **(B)** Number of platform crossings in each group on day 6. **(C)** Representative swimming traces of mice in each group on day 5 of the place navigation test. **(D)** Mean escape latency of mice in each group over days 1–5. ###*P* < 0.001 vs. control group; *P < 0.05, **P < 0.01, ***P < 0.001 vs. model group.

The experiment lasted for 6 consecutive days. The results showed that the mean escape latency of mice in all groups gradually decreased over the training days. Compared with the model group, the mean escape latency in the GFP-high group was significantly reduced on days 2–5, in the donepezil group on days 3–5, and in the GFP-medium group on day 5. On day 6 (probe trial), the number of platform crossings in the GFP-high group and the donepezil group was significantly increased compared with the model group (Figure 2).

### 3.3 Transcriptomics

**FIGURE 3:**
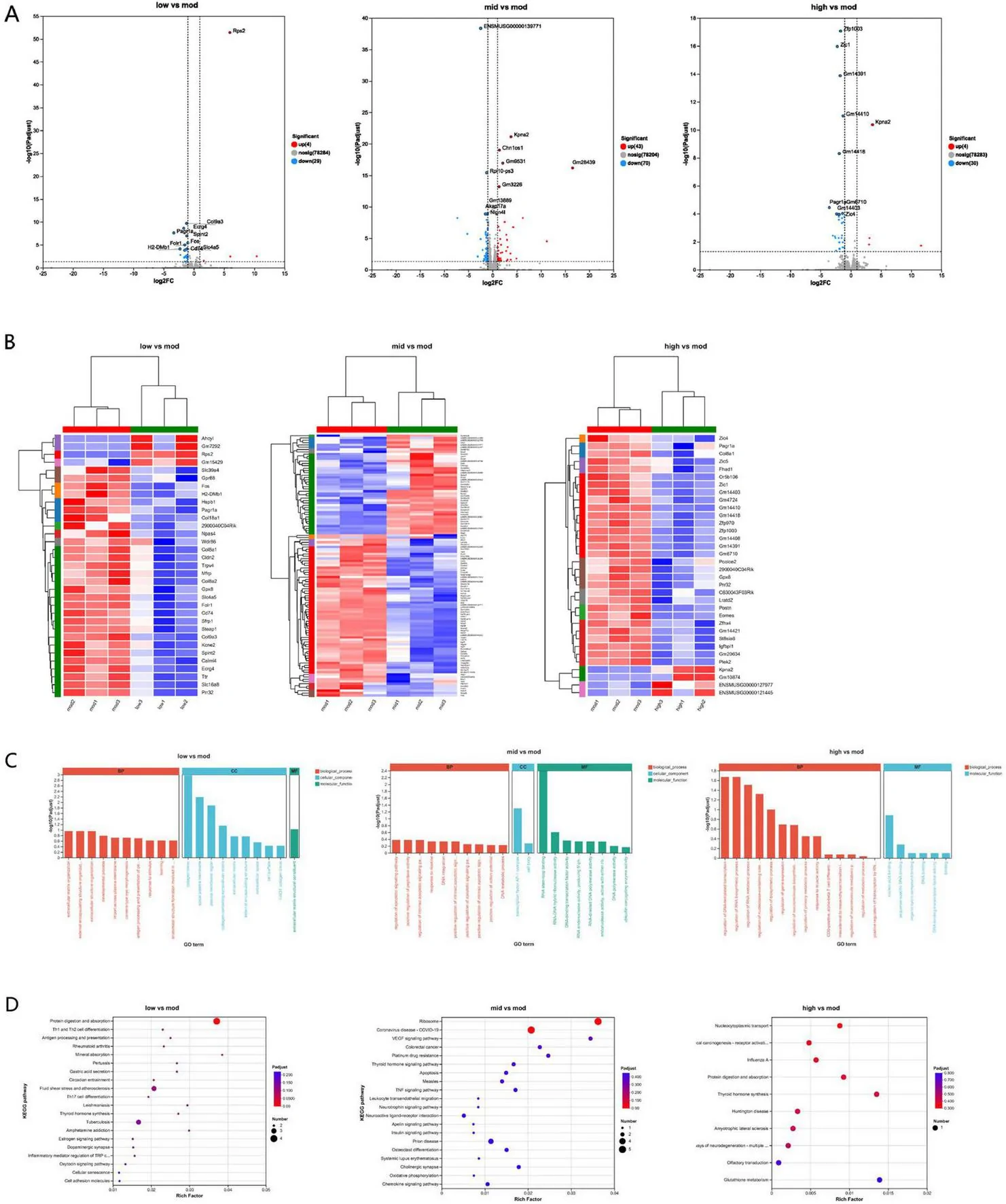
Transcriptomic analysis. **(A)** Volcano plots of differentially expressed genes (DEGs) between the GFP-low, GFP-medium, and GFP-high groups versus the model group. **(B)** Clustered heatmaps of DEGs between the GFP-low, GFP-medium, and GFP-high groups versus the model group. **(C)** GO enrichment analysis of DEGs in the GFP-low, GFP-medium, and GFP-high groups versus the model group. **(D)** KEGG pathway enrichment analysis of DEGs in the GFP-low, GFP-medium, and GFP-high groups.

Transcriptomic analysis was performed on the model, GFP-low, GFP-medium, and GFP-high groups (*n* = 3 per group). As shown in the volcano plots and heatmaps (Figures 3A, B), compared with the model group, 33 differentially expressed genes (DEGs) were identified in the GFP-low group, including 4 upregulated and 29 downregulated genes; 113 DEGs (43 upregulated and 70 downregulated) in the GFP-medium group; and 34 DEGs (4 upregulated and 30 downregulated) in the GFP-high group. These results indicated that the GFP-medium group exhibited the most pronounced modulation of gene expression.

Notably, compared with the model group, GFP treatment significantly upregulated Kpna2. Previous studies have shown that Kpna2 overexpression promotes STAT3 phosphorylation, which subsequently upregulates VEGF and ANGPT2, thereby facilitating angiogenesis under hypoxic conditions (Jia et al., 2022). In addition, Postn, a marker of reactive astrocytes and neuroinflammation, was significantly downregulated following treatment (Wang et al., 2024). GO enrichment analysis (Figure 3C) revealed that the DEGs were significantly enriched in biological processes related to inflammatory responses and oxidative stress. KEGG pathway analysis (Figure 3D) indicated that these genes were primarily involved in signaling pathways associated with inflammation, apoptosis, and BBB integrity, including the VEGF signaling pathways, TNF signaling pathways, and apoptosis signaling pathways. Collectively, these findings suggest that GFP may ameliorate AD-like cognitive impairment through multiple mechanisms, including attenuation of inflammatory responses, inhibition of apoptosis, and protection of the BBB.

### 3.4 Gut microbiota analysis

**FIGURE 4:**
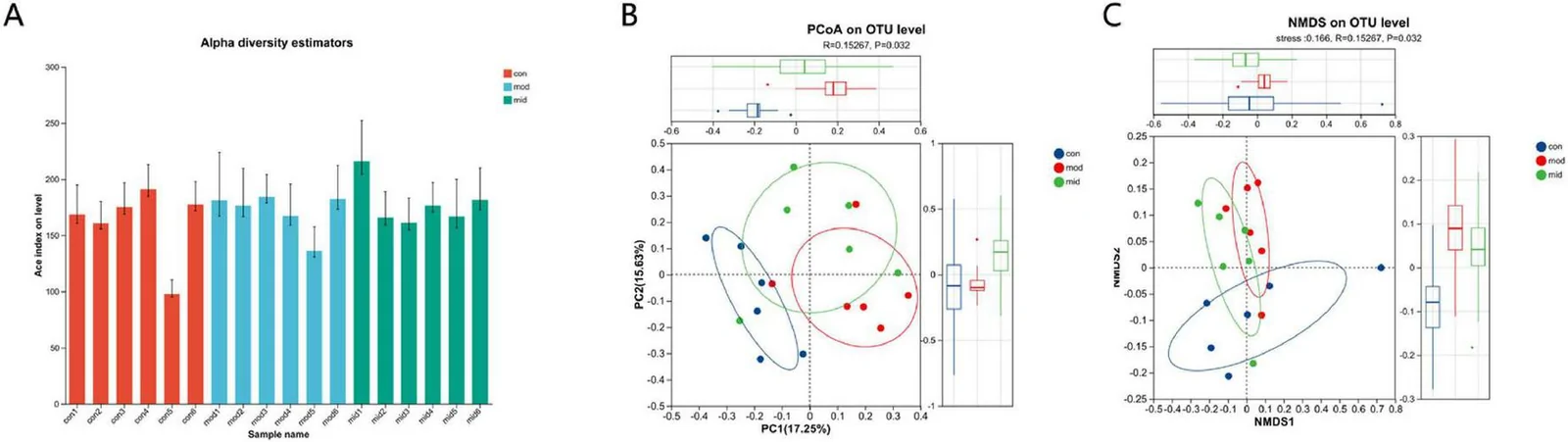
**(A)** Ace index. **(B)** PCoA analysis. **(C)** NMDS analysis.

To determine the effects of GFP on the gut microbiota of mice with AD-like cognitive impairment, 16S rRNA gene sequencing was performed on 18 fecal samples collected from the control group (*n* = 6), model group (*n* = 6), and GFP-low group (*n* = 6). The GFP-medium group, which corresponds to the clinical equivalent dose, was selected among the three GFP dose groups for analysis, as it more closely reflects clinical practice and provides a more reliable reference for future clinical trials. The Ace index indicated that GFP treatment did not significantly affect the microbial richness in model mice (Figure 4A). Principal coordinate analysis (PCoA) and non-metric multidimensional scaling (NMDS) analyses revealed distinct clustering and separation among the control, model, and GFP groups (Figures 4B,C).

**FIGURE 5:**
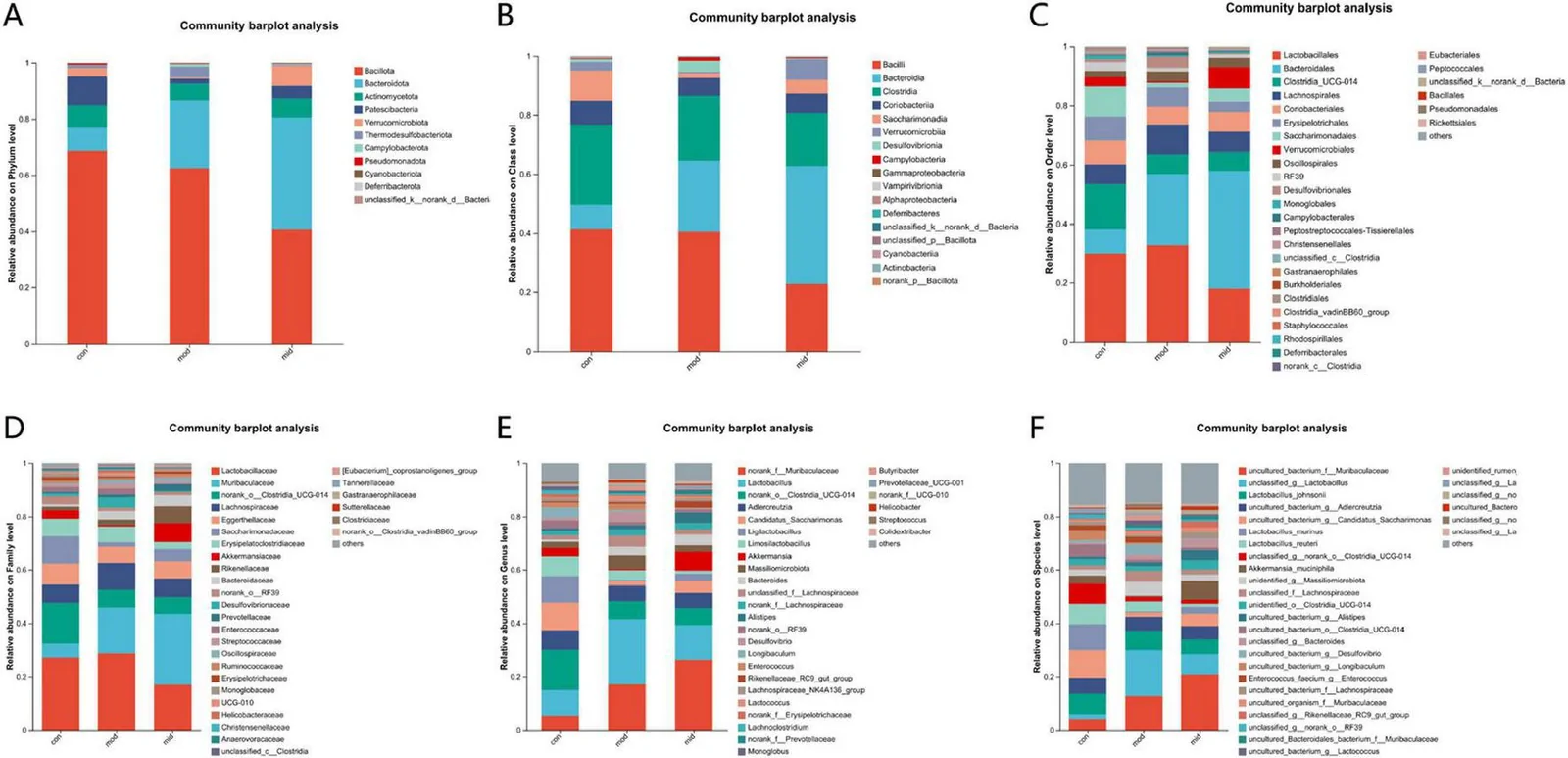
Structural changes in the microbiota at the phylum, class, order, family, genus, and species levels. **(A)** Phylum level, **(B)** class level, **(C)** order level, **(D)** family level, **(E)** genus level, **(F)** species level.

**FIGURE 6:**
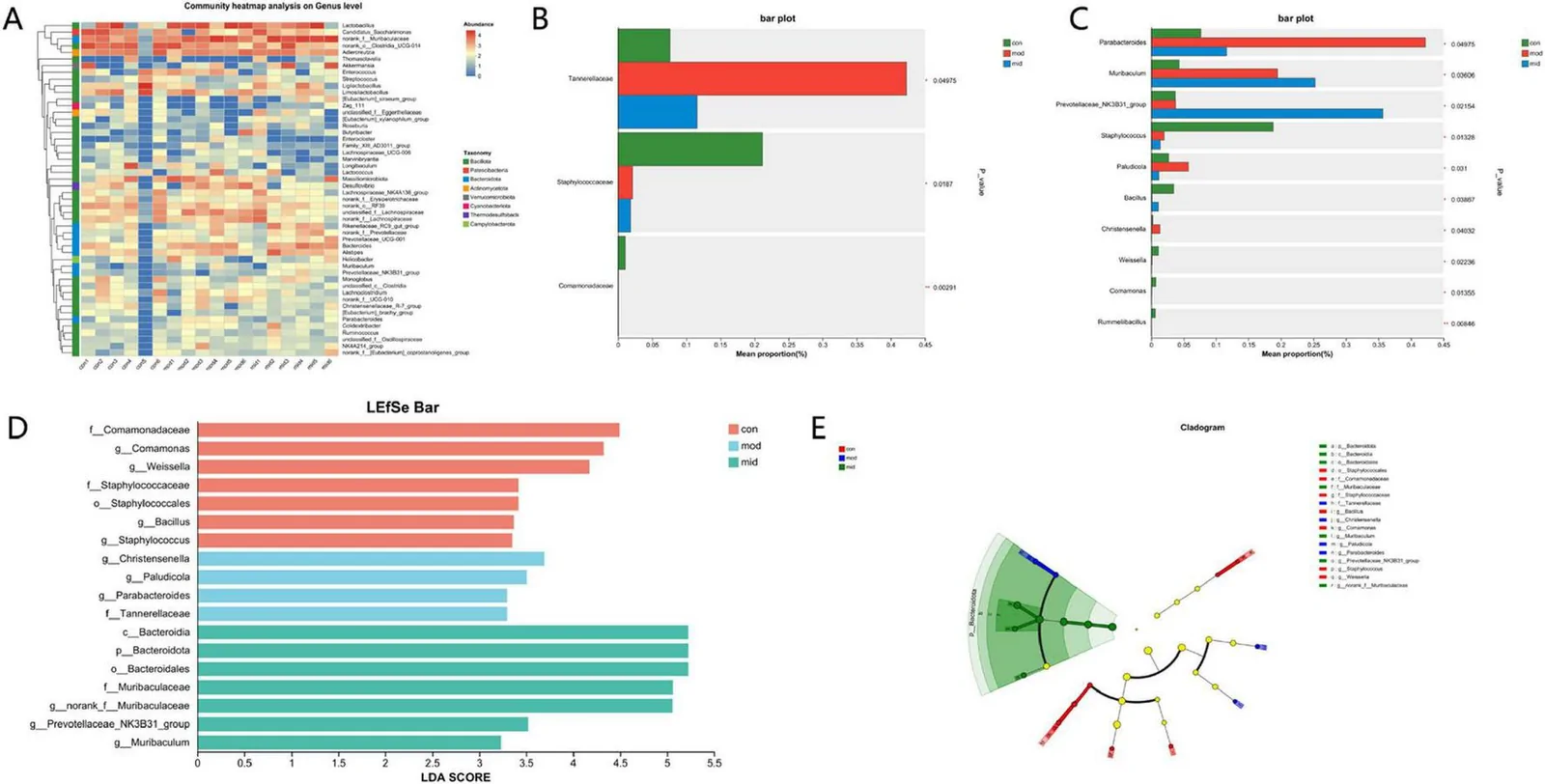
**(A)** Community heatmap; **(B,C)** Kruskal-Wallis test; **(D,E)** LEFSe multi-level species discriminant analysis.

To determine the effects of GFP on the microbial composition and structure, the relative abundance of microbiota was analyzed at the phylum, class, order, family, genus, and species levels (Figures 5A–F). The results showed that compared with the control group, the model group exhibited decreased relative abundances of Actinomycetota, Verrucomicrobiota, Coriobacteriia, Saccharimonadia, Lactobacillales, Lachnospirales, Eggerthellaceae, Saccharimonadaceae, *Ligilactobacillus*, and *Akkermansia*, whereas these taxa were increased in the GFP group. In contrast, the relative abundances of Thermodesulfobacteriota, Campylobacterota, Desulfovibrionia, Lachnospirales, Lachnospiraceae, and Desulfovibrionaceae were increased in the model group compared with the normal group, but decreased following GFP treatment. The community heatmap is shown in Figure 6A.

To identify the bacterial taxa that may play key roles, intergroup comparisons at the family and genus levels were further performed using the Kruskal–Wallis test (Figures 6B–E). The results showed that the relative abundances of Tannerellaceae, Parabacteroides, Paludicola, and Christensenella were increased in the model group compared with the control group, but decreased in the GFP group. In contrast, the relative abundance of Bacillus was decreased in the model group compared with the control group, but increased in the GFP group. Linear discriminant analysis effect size (LEfSe) was used to identify unique biomarkers in each group (Figures 6B–E). The results revealed that the biomarkers of the control group included Comamonadaceae, Comamonas, and Weissella, while those of the model group included Christensenella and Paludicola. The biomarkers of the GFP group included Bacteroidia and Bacteroidota. These findings suggest that GFP may ameliorate cognitive impairment in AD-like mice, at least in part, by modulating the gut microbiota.

### 3.5 Metabolomics

**FIGURE 7:**
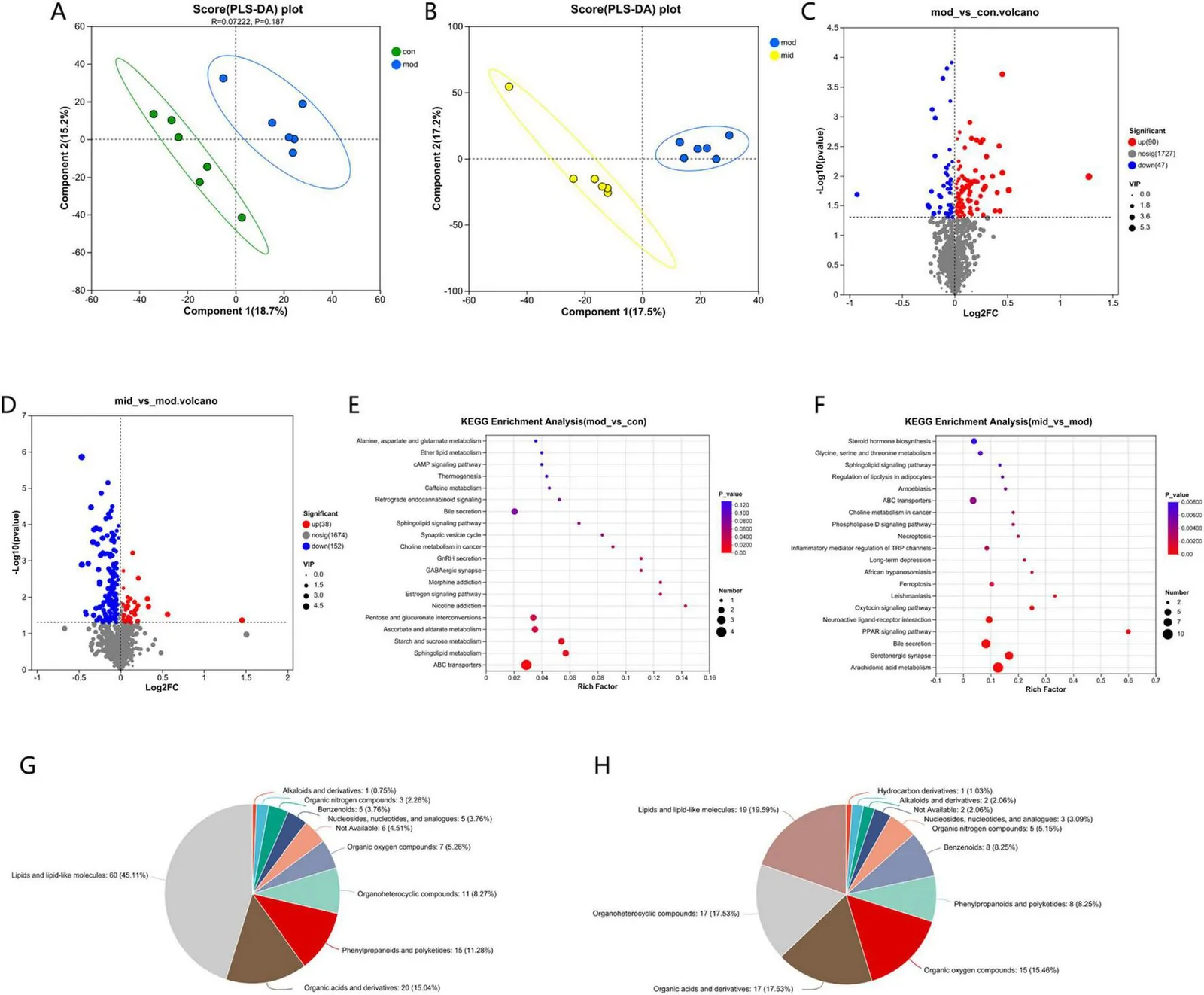
Serum metabolomics analysis. **(A)** PLS-DA score plot (model vs. control); **(B)** PLS-DA score plot (GFP -medium vs. model); **(C)** volcano plot of differential metabolites (model vs. control); **(D)** volcano plot of differential metabolites (GFP-medium vs. model); **(E)** bubble plot (model vs. control); **(F)** bubble plot (GFP-medium vs. model); **(G)** pie chart of HMDB compound classification (model vs. control); **(H)** pie chart of HMDB compound classification (GFP-medium vs. model).

To determine the effects of GFP on serum metabolites in model mice, untargeted serum metabolomics analysis was performed on 18 serum samples collected from the control group (*n* = 6), model group (*n* = 6), and GFP-medium group (*n* = 6). The medium dose (clinical equivalent dose) was selected as the treatment group for analysis. Partial least squares discriminant analysis (PLS-DA) revealed (Figures 7A,B) that the model group exhibited significant metabolic disturbances, which were partially restored following GFP treatment. Differential metabolite analysis (Figures 7C,D) showed that 137 differential metabolites (90 upregulated, 47 downregulated) were identified between the model and control groups, and 190 differential metabolites (38 upregulated, 152 downregulated) between the GFP and model groups. KEGG analysis revealed that the differential metabolites between the model and control groups were enriched in pathways such as ABC transporters and sphingolipid metabolism, while the differential metabolites between the GFP and model groups were enriched in pathways including serotonergic synapse and arachidonic acid metabolism (Figures 7E,F). Among these, lipids and lipid-like molecules accounted for 22.6% of the differentially abundant metabolites between the GFP and model groups. GFP significantly affected the relative abundance of serum metabolites, particularly terpene lipids (Figures 7G,H). These findings suggest that GFP may improve AD-like cognitive impairment by regulating the synthesis of lipids and lipid-like molecules.

### 3.6 H&E staining

**FIGURE 8:**
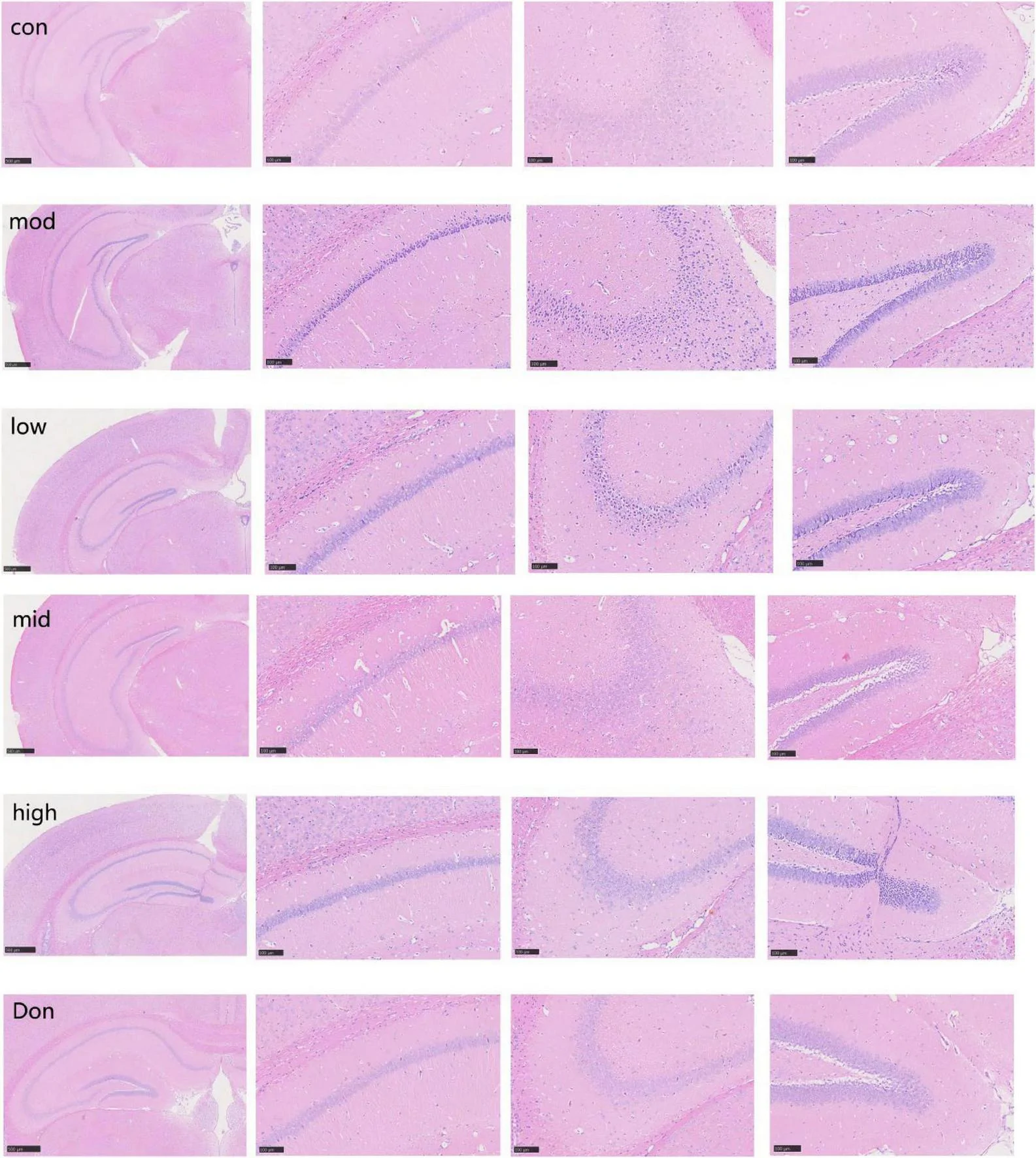
H&E staining of each group. Con, control group; Mod, model group; Low, GFP-low group; Mid, GFP-medium group; High, GFP-high group; Don, donepezil group.

The effects of GFP on hippocampal histopathology in aged mice were evaluated by H&E staining. As shown in Figure 8, the hippocampal neurons in the control group exhibited a regular and well-organized arrangement, with spherical morphology, intact cell membranes and nuclei, and no obvious swelling or necrosis. In contrast, the model group displayed marked histopathological abnormalities, including disorganized neuronal arrangement, heterogeneous cell size and structure, and a significant reduction in neuronal number. Treatment with GFP or donepezil partially ameliorated these morphological alterations, suggesting that GFP and donepezil preserved neuronal integrity and attenuated hippocampal histological damage in model mice.

### 3.7 Network pharmacological analysis

**FIGURE 9:**
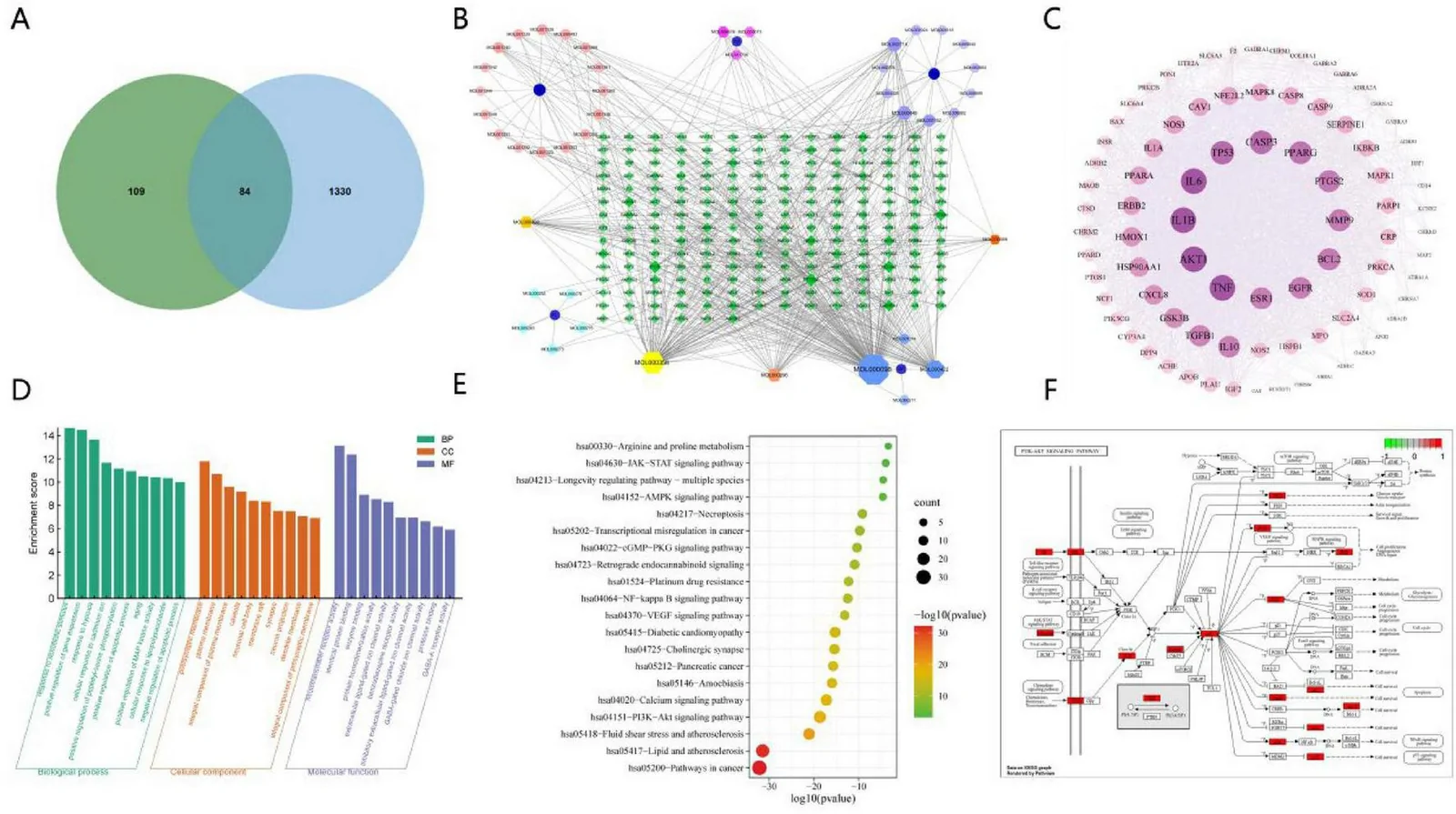
Network pharmacological analysis. **(A)** Venn diagram of GFP targets and AD-related targets; **(B)** compound-target network of bioactive compounds in GFP and AD-related targets; **(C)** PPI network of overlapping targets between GFP and AD; **(D)** GO enrichment analysis of overlapping targets; **(E)** KEGG pathway enrichment analysis of overlapping targets; **(F)** representative KEGG pathway maps of selected signaling pathways.

Network pharmacology analysis was employed to investigate the mechanisms underlying GFP in the treatment of AD-like cognitive impairment. A total of 76 eligible compounds and 223 potential targets of the active ingredients were screened, and 1414 disease-related targets for AD were identified, yielding 84 overlapping targets between the drug and the disease. PPI network analysis suggested that AKT1, IL6, and TNF may play important roles in the prevention and treatment of AD. GO enrichment analysis revealed that the biological processes involved included positive regulation of protein phosphorylation and regulation of apoptotic signaling pathways; cellular components mainly involved the postsynaptic membrane and dendrites; and molecular functions were primarily associated with neurotransmitter receptor activity and G protein-coupled amine receptor activity. The major KEGG pathways identified were the VEGF signaling pathway and the PI3K/AKT signaling pathway. Collectively, these findings suggest that the mechanisms by which GFP ameliorates AD-like cognitive impairment may be associated with angiogenesis and inflammatory responses (Figure 9).

### 3.8 IHC analysis

**FIGURE 10:**
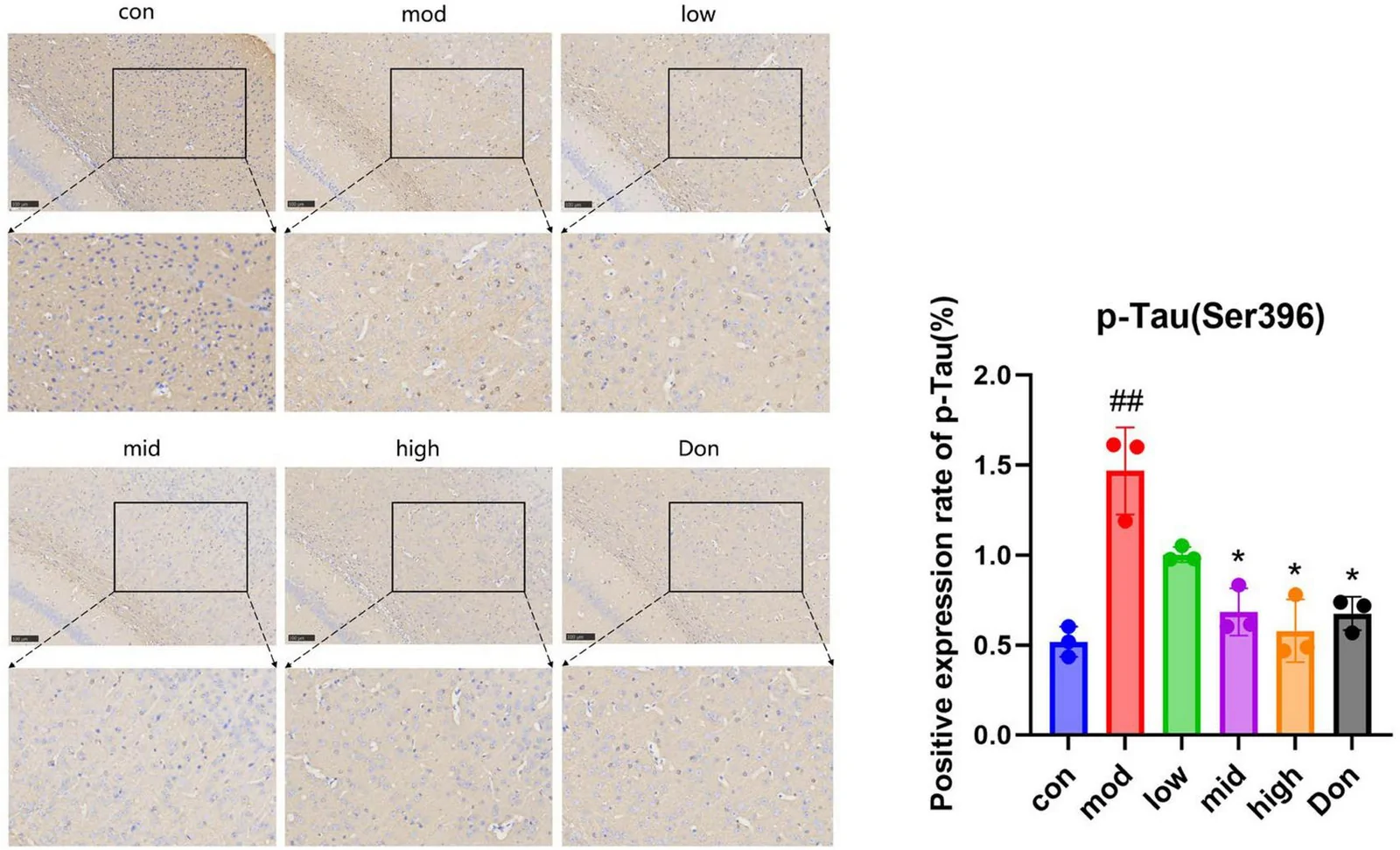
IHC of p-Tau (Ser396) in each group. Con, control group; Mod, model group; Low, GFP-low group; Mid, GFP-medium group; High, GFP-high group; Don, donepezil group. ^##^*P* < 0.01 vs. control group; *P < 0.05 vs. model group.

As shown in Figure 10, the expression of p-Tau (Ser396) in the cerebral cortex was significantly increased in the model group compared with the control group (*P* < 0.01). Compared with the model group, the expression of p-Tau (Ser396) was significantly downregulated in the GFP-medium group, the GFP-high group, and the donepezil group (*P* < 0.05).

## 4 Discussion

This study employed integrated approaches, including transcriptomics, metabolomics, gut microbiota analysis, and network pharmacology, to investigate the effects and mechanisms of GFP on D-gal-induced AD-like cognitive impairment in aged mice. The results showed that GFP improved learning and memory functions in aged mice, alleviated neuronal damage in the hippocampus, and reduced the abnormal accumulation of p-Tau (Ser396). Furthermore, GFP exerted certain effects on hippocampal gene expression, gut microbiota, and metabolites in aged mice. It is speculated that its cognitive-improving effects may be mediated through the regulation of relevant pathways, modulation of gut microbiota, and influence on the synthesis of lipids and lipid-like molecules. These findings provide scientific evidence and new insights for the application of GFP in improving AD-like cognitive impairment.

Through the Morris water maze, H&E staining, and IHC, we found that GFP intervention improved cognitive impairment in aged mice, alleviated hippocampal neuronal damage, and reduced the abnormal accumulation of p-Tau (Ser396) in the cortex. To identify the potential mechanisms by which GFP ameliorates AD-like cognitive impairment, network pharmacology and transcriptomic analyses were employed to explore key targets and pathways. The results showed that Kpna2, a gene important for promoting angiogenesis and neuroprotection, was significantly upregulated in the GFP group, while Postn, a marker driving neuroinflammation, was significantly downregulated. Signaling pathways related to inflammatory responses, the BBB, and apoptosis played important roles in this process. These findings suggest that the key mechanisms underlying the ameliorative effects of GFP on AD-like cognitive impairment may be associated with the attenuation of neuroinflammatory responses and the repair of the BBB.

Gut microbiota analysis has been widely used in studies on inflammation-related diseases (Dong et al., 2023). Gut microbiota dysbiosis has been proposed as one of the pathological bases of AD (Chen et al., 2022). In this study, we investigated the effects of GFP on the abundance and composition of gut microbiota in mice with AD-like cognitive impairment by observing changes in the gut microbiota. The results showed that GFP exerted a beneficial effect on the gut microbiota in aged mice.

Studies have found that microbiota dysbiosis may be one of the factors contributing to neuroinflammatory processes in AD (Angelucci et al., 2019). Lactobacillales are the predominant probiotics in the gut and exert significant effects on neurological diseases (Wang et al., 2024; Willyard, 2021). Increasing the abundance of Lactobacillales has been shown to exert positive effects on cognition and pathological changes in AD model mice (Di Salvo et al., 2024; Hamid and Zahid, 2023; Song et al., 2022); for example, oat protein hydrolysate can restore Lactobacillus abundance and regulate the gut-serum-brain axis to prevent AD-like symptoms (Rafique et al., 2023). Lactobacillus has been reported to ameliorate cognitive impairment and pathological changes in SAMP8 mice, including neuronal damage, Aβ and Tau pathology, and neuroinflammation (Xiao-Hang et al., 2024). Desulfovibrionia possess the ability to reduce sulfate to hydrogen sulfide, and the accumulation of hydrogen sulfide can induce chronic inflammation and disrupt the balance between cell proliferation and apoptosis (Lindenberg et al., 2019). Tannerellaceae, as oral pathogens, exhibit a high presence at periodontitis lesion sites, suggesting their role in promoting inflammation (Chukkapalli et al., 2015). In sleep-deprived C57BL/6J mice, the abundance of Tannerellaceae in the gut was positively correlated with the levels of pro-inflammatory cytokines (Zhang et al., 2023). *Akkermansia* is a mucin-degrading bacterium, and studies have found that *Akkermansia* and its metabolite propionic acid can maintain mitochondrial fission and autophagic homeostasis in the pathological process of AD (Wang et al., 2025).

Metabolomics analysis revealed that lipids and lipid-like molecules were the most significantly differential metabolites between the GFP group and the model group. GFP intervention exerted a significant effect on serum metabolites, particularly on the relative abundance of terpenoid lipids. Terpenoid lipids serve as a critical bridge connecting primary and secondary metabolism. Terpenoid lipids are diverse in both variety and function, and multiple steroids and terpenoid-derived phytochemicals have been reported to play important roles in AD-related neuroinflammatory pathways (Kabir et al., 2021). For instance, ginkgolide B exerts a protective effect against ischemic stroke by upregulating the expression of silent mating type information regulation 2 homolog 1 (SIRT1), anti-apoptotic proteins, and heme oxygenase 1 (Nabavi et al., 2015). Ginsenoside Rg3 and its derivatives possess sufficient BBB penetrating ability, thereby exerting therapeutic effects on neurological diseases (Kim et al., 2014).

Limitations of this study: In the gut microbiota and metabolomics analyses, only the GFP-medium group was selected for analysis, with only 3–6 mice per group. Although the mice were randomly selected, the small sample size may still introduce bias into the findings and limit the generalizability of the results. Future studies should expand the sample size, include analyses across all subgroups, and incorporate additional experimental validation and spatial transcriptomics to further explore the mechanisms underlying the effects of GFP on AD-like cognitive impairment. In addition, this study used D-gal to establish an aged mouse model, which only mimics the risk factors for AD onset and does not fully recapitulate the core pathological hallmarks of AD. Future research could employ APP/PS1 transgenic mice to further investigate the effects of GFP on AD-specific pathological changes.

## 5 Conclusion

This study employed transcriptomics, metabolomics, and gut microbiota analysis to investigate the mechanisms underlying the effects of GFP on AD-like cognitive impairment. The results showed that GFP improved cognitive impairment in aged mice to a certain extent and alleviated gut microbiota dysbiosis and metabolic disorders. Transcriptomics and network pharmacology analyses suggested that the Kpna2 gene plays an important role in this process, and that regulation of the VEGF signaling pathway and the PI3K/AKT pathway, along with the promotion of angiogenesis and attenuation of inflammatory responses, may represent the potential mechanisms underlying its cognitive-improving effects. Collectively, our findings indicate that GFP may serve as a potential therapeutic approach for ameliorating AD-like cognitive impairment through the regulation of gut microbiota, improvement of metabolic disorders, promotion of angiogenesis, and reduction of inflammatory responses.

## Data Availability

The datasets presented in this study can be found in online repositories. The names of the repository/repositories and accession number(s) can be found below: https://www.ncbi.nlm.nih.gov/, PRJNA1428548.

## Footnotes

1
https://github.com/OpenGene/fastp

2
http://ccb.jhu.edu/software/hisat2/index.shtml

3
http://deweylab.github.io/RSEM/

4
http://bioconductor.org/packages/stats/bioc/DESeq2/

5
https://github.com/tanghaibao/GOatools

6
http://kobas.cbi.pku.edu.cn/home.do

7
https://cloud.majorbio.com

8
https://github.com/OpenGene/fastp

9
http://www.cbcb.umd.edu/software/flash

10
http://drive5.com/usearch/

11
http://rdp.cme.msu.edu/

12
http://www.hmdb.ca/

13
https://metlin.scripps.edu/
