# Supplementary material for: The molecular mechanisms of Guizhi Fuling Pills in ameliorating Alzheimer’s disease-like cognitive impairment: insights from transcriptomics, metabolomics, and gut microbiome
Source: Front Aging Neurosci. 2026 Aug 13;18:1839445. doi: 10.3389/fnagi.2026.1839445 (PMC13518511; doi:10.3389/fnagi.2026.1839445)
Supplement: Supplementary file 1 [file Table_1.docx]

Supplementary Table HR-LC-MS technology identified 473 components in GFP

| NO. | Name | Formula | Annot. DeltaMass [ppm] | mzVault Best Match |
| --- | --- | --- | --- | --- |
| 1 | o-Veratraldehyde | C9 H10 O3 | 10.44 | 42.3 |
| 2 | Citric acid | C6 H8 O7 | 9.7 | 91 |
| 3 | Cinnamaldehyde | C9 H8 O | 11.48 | 91.4 |
| 4 | Albiflorin | C23 H28 O11 | 11.25 | 80.5 |
| 5 | Ethyl gallate | C9 H10 O5 | 9.41 | 90.1 |
| 6 | (S)-MALATE | C4 H6 O5 | 4.06 | 94.3 |
| 7 | 9-Methoxy-2,2-dimethyl-2,6-dihydro-pyrano[3,2-c]quinolin-5-one | C15 H15 N O3 | 1.28 | 50.8 |
| 8 | NCGC00380749-01_C30H32O12_{(1R,2S,3R,5R,6R,8S)-3-[(6-O-Benzoyl-beta-D-glucopyranosyl)oxy]-6-hydroxy-8-methyl-9,10-dioxatetracyclo[4.3.1.0~2,5~.0~3,8~]dec-2-yl}methyl benzoate | C30 H32 O12 | 37638.23 | 81 |
| 9 | THEOBROMINE | C7 H8 N4 O2 | 3.22 | 72.5 |
| 10 | Ferulaldehyde | C10 H10 O3 | 11.3 | 47.5 |
| 11 | Paeoniflorin | C23 H28 O11 | 35470.86 | 89.6 |
| 12 | Steviol | C20 H30 O3 | 2.99 | 72.7 |
| 13 | NCGC00381007-01_C21H23NO8_(5S,8S,9R)-8-Benzoyl-2-[(1S,2S,3Z)-1,2-dihydroxy-3-hexen-1-yl]-8,9-dihydroxy-3-methyl-1-oxa-7-azaspiro[4.4]non-2-ene-4,6-dione | C21 H23 N O8 | 10.94 | 43.3 |
| 14 | Cantharidin | C10 H12 O4 | 10.75 | 54 |
| 15 | Carylophyllene Oxide | C14 H22 O | -92369.85 | 43.7 |
| 16 | Meglutol | C6 H10 O5 | 11.15 | 33.9 |
| 17 | Protocatechualdehyde | C7 H6 O3 | 4.73 | 90.4 |
| 18 | Veratric acid | C9 H10 O4 | 11.68 | 38.3 |
| 19 | "3,4-DIHYDROXYBENZOATE" | C7 H6 O4 | 6.46 | 96.5 |
| 20 | SUCCINATE | C4 H6 O4 | 2.98 | 94.7 |
| 21 | falcarindiol | C17 H24 O2 | -123091.73 | 46.3 |
| 22 | Pyrogallol | C6 H6 O3 | 3.81 | 90 |
| 23 | Eremanthin | C15 H18 O2 | 21586.94 | 60.3 |
| 24 | (4aS)-6,7-dihydroxy-1,1,4a-trimethyl-3,4,10,10a-tetrahydro-2H-phenanthren-9-one | C17 H22 O3 | 2.94 | 66.4 |
| 25 | 4'-ethenyl-2'-hydroxy-1,4',4a-trimethyl-5-oxospiro[2,3,4,7,8,8a-hexahydronaphthalene-6,1'-cyclopentane]-1-carboxylic acid | C20 H30 O4 | 2.99 | 43.4 |
| 26 | Ethylparaben | C9 H10 O3 | 10.71 | 30.3 |
| 27 | Sophocarpine | C15 H22 N2 O | -5 | 32.9 |
| 28 | (4S,5Z,6S)-4-(2-methoxy-2-oxoethyl)-5-[2-[(E)-3-phenylprop-2-enoyl]oxyethylidene]-6-[(2S,3R,4S,5S,6R)-3,4,5-trihydroxy-6-(hydroxymethyl)oxan-2-yl]oxy-4H-pyran-3-carboxylic acid | C12 H8 O4 | -212945.98 | 35 |
| 29 | Rutaecarpine | C18 H13 N3 O | -6.43 | 39.5 |
| 30 | MLS001146949-01!18-BETA-GLYCYRRHETINIC ACID471-53-4 | C30 H46 O4 | 11.86 | 30.5 |
| 31 | Retapamulin | C30 H47 N O4 S | 0.56 | 44.9 |
| 32 | Corosolic acid | C30 H48 O4 | 11.31 | 44 |
| 33 | NCGC00385350-01_C20H26N2O2_(17R,21alpha)-Ajmalan-17,21-diol | C20 H26 N2 O2 | -4.54 | 50.6 |
| 34 | Pesticide3_Pirimicarb_C11H18N4O2_2-(Dimethylamino)-5,6-dimethyl-4-pyrimidinyl dimethylcarbamate | C11 H18 N4 O2 | 6.76 | 47.2 |
| 35 | "(8R,9S,10R,13S,14S,17S)-17-hydroxy-10,13,17-trimethyl-2,6,7,8,9,11,12,14,15,16-decahydro-1H-cyclopenta[a]phenanthren-3-one" | C20 H30 O2 | 4.09 | 68.8 |
| 36 | Kahweol | C20 H26 O3 | 4.93 | 70.5 |
| 37 | NCGC00180814-02_C20H28O3_2-Naphthalenecarboxylic acid, 1-[2-(3-furanyl)ethyl]-1,2,3,4,4a,7,8,8a-octahydro-1,4a,5-trimethyl-, (1R,2R,4aR,8aR)- | C20 H28 O3 | 4.05 | 82.9 |
| 38 | Pesticide6_Metconazole_C17H22ClN3O_5-(4-Chlorophenylmethyl)-2,2-dimethyl-1-(1H-1,2,4-triazol-1-ylmethyl)cyclopentanol | C17 H22 Cl N3 O | -56432.55 | 34.7 |
| 39 | 5-OXO-L-PROLINE | C5 H7 N O3 | 4.32 | 89.2 |
| 40 | NCGC00381312-01!5-[10-(3,5-dihydroxyphenyl)decyl]benzene-1,3-diol | C22 H30 O4 | 3.65 | 43.6 |
| 41 | Neoruscogenin | C27 H40 O4 | -51318.99 | 43.7 |
| 42 | Ginkgolic acid C17-1 | C24 H38 O3 | -58726.09 | 46.3 |
| 43 | p-Hydroxybenzaldehyde | C7 H6 O2 | 3.44 | 87.5 |
| 44 | Alterperylenol | C20 H14 O6 | -62415.42 | 65.4 |
| 45 | NCGC00169374-02_C23H28O11_7-Oxatricyclo[4.3.0.0~3,9~]nonan-8-one, 9-[(benzoyloxy)methyl]-1-(beta-D-glucopyranosyloxy)-4-hydroxy-6-methyl-, (1R,3R,4R,6S,9S)- | C23 H28 O11 | 6.68 | 33.5 |
| 46 | NCGC00381398-01_C17H26O4_6-[(2E,4E)-4,6-Dimethyl-2,4-octadienoyl]-2,3-dihydroxy-2-methylcyclohexanone | C17 H26 O4 | -135941.55 | 30 |
| 47 | 1-METHYLADENOSINE | C11 H15 N5 O4 | 7.09 | 84.6 |
| 48 | 2-METHYLMALEATE | C5 H6 O4 | 4.82 | 88.3 |
| 49 | NCGC00169089-04_C19H24O4_Benzaldehyde, 6-[(3E,5E,7S)-5,7-dimethyl-2-oxo-3,5-nonadien-1-yl]-2,4-dihydroxy-3-methyl- | C19 H24 O4 | -56962.33 | 62.5 |
| 50 | 1,4-dihydroxy-6,6,9a-trimethyl-4,5,5a,7,8,9-hexahydro-1H-benzo[e][2]benzofuran-3-one | C15 H22 O4 | 82598.72 | 60.8 |
| 51 | Celastrol | C29 H38 O4 | 6.22 | 40.5 |
| 52 | NCGC00380586-01!methyl 4,5,6-trihydroxy-3-(3-methoxy-3-oxoprop-1-en-2-yl)oxycyclohexene-1-carboxylate | C12 H16 O8 | 59115.03 | 32.3 |
| 53 | Pesticide5_Metribuzin_C8H14N4OS_4-Amino-6-(2-methyl-2-propanyl)-3-(methylsulfanyl)-1,2,4-triazin-5(4H)-one | C8 H14 N4 O S | -9.82 | 42.4 |
| 54 | Ethyl 3,4-dihydroxybenzoate 3,4 | C9 H10 O4 | 9.5 | 73.4 |
| 55 | NCGC00385387-01_C16H28O7_4-(Hydroxymethyl)-1-isopropyl-3-cyclohexen-1-yl beta-D-glucopyranoside | C16 H28 O7 | 66184.87 | 79.7 |
| 56 | Pesticide3_Chlorotoluron_C10H13ClN2O_Urea, N'-(3-chloro-4-methylphenyl)-N,N-dimethyl- | C10 H13 Cl N2 O | -1.16 | 48.6 |
| 57 | 4-hydroxyphenylacetic acid | C8 H8 O3 | 6.53 | 70.2 |
| 58 | makaluvamine C | C11 H12 N3 O | -4974.67 | 30.6 |
| 59 | NCGC00347834-02_C20H30O5_6,10a-Dihydroxy-4-(hydroxymethyl)-4,7,11b-trimethyl-2,3,4,4a,5,6,6a,7,10a,11,11a,11b-dodecahydrophenanthro[3,2-b]furan-9(1H)-one | C20 H30 O5 | -51424.85 | 38.5 |
| 60 | Hypoxanthine | C5 H4 N4 O | -5.38 | 49.3 |
| 61 | MaprotilineHCl | C20 H24 Cl N | -111670.54 | 69.1 |
| 62 | Higenamine | C16 H17 N O3 | -2.54 | 47 |
| 63 | Azelaic acid | C9 H16 O4 | 9.39 | 90.6 |
| 64 | 4-amino-5-(5-hydroxy-2',6-dimethyl-5'-prop-1-en-2-ylspiro[7-oxabicyclo[4.1.0]heptane-3,1'-cyclopentane]-2-yl)oxy-5-oxopentanoic acid | C20 H31 N O6 | 57665.18 | 32.2 |
| 65 | NCGC00170025-02!N-(1-hydroxy-3-phenylpropan-2-yl)benzamide | C16 H17 N O2 | -4.29 | 38.3 |
| 66 | Quinic acid | C7 H12 O6 | 11.13 | 68.8 |
| 67 | NCGC00385951-01!1,3,6-trihydroxy-4,5-dimethoxy-10-methylacridin-9-one | C16 H15 N O6 | 10.99 | 88.2 |
| 68 | Aphidicolin | C20 H34 O4 | 3.8 | 30.6 |
| 69 | "(6S,8R,9S,10R,13S,14S,17R)-17-acetyl-17-hydroxy-6,10,13-trimethyl-2,6,7,8,9,11,12,14,15,16-decahydro-1H-cyclopenta[a]phenanthren-3-one" | C22 H32 O3 | -63853.64 | 72.6 |
| 70 | NCGC00385492-01_C14H16O3_(3R,3aR)-3-(3-Furyl)-3a,7-dimethyl-3a,4,5,6-tetrahydro-2-benzofuran-1(3H)-one | C14 H16 O3 | -77599.87 | 30 |
| 71 | NCGC00347365-02!5-hydroxy-2,2-dimethyl-10-(2-methylbut-3-en-2-yl)pyrano[3,2-g]chromen-8-one | C19 H20 O4 | -1.57 | 46.4 |
| 72 | NCGC00380393-01_C15H20O4_Pentaleno[1,6a-c]pyran-9-carboxylic acid, 1,3,4,5,6,7,7a,9a-octahydro-4,6,6-trimethyl-3-oxo-, (4S,4aR,7aS,9aR)- | C15 H20 O4 | -458385.19 | 72.6 |
| 73 | NCGC00385002-01_C22H32O7_Butanoic acid, 3-methyl-, (3alpha,8alpha,12xi)-15-(acetyloxy)-12,13-epoxy-3-hydroxytrichothec-9-en-8-yl ester | C22 H32 O7 | 6.12 | 33.4 |
| 74 | NCGC00169660-02_C22H30O3_2-Hydroxy-6-[(8Z,11Z)-8,11,14-pentadecatrien-1-yl]benzoic acid | C22 H30 O3 | 3.76 | 35.5 |
| 75 | Eicosanoids_ResolvinD1_C22H32O5 |  |  |  |
| 76 | NCGC00179896-02_C16H22O8_beta-D-Glucopyranoside, 4-[(1E)-3-hydroxy-1-propen-1-yl]-2-methoxyphenyl | C16 H22 O8 | 64261.52 | 85 |
| 77 | Methyl gallate | C8 H8 O5 | 97873.18 | 87.8 |
| 78 | Westcort | C26 H38 O6 | 0.67 | 38.6 |
| 79 | D-(-)-quinic acid | C7 H12 O6 | 10.77 | 86.2 |
| 80 | NCGC00384746-01!6-(2,3-dihydroxy-3-methylbutyl)-7-methoxy-3-(2-methylbut-3-en-2-yl)chromen-2-one | C20 H26 O5 | 4.77 | 42.2 |
| 81 | "Mirtazapine (Remeron, Avanza)" | C17 H19 N3 | -8.91 | 53 |
| 82 | Maleic acid | C4 H4 O4 | 2.85 | 83.1 |
| 83 | Levofloxacin (Levaquin) | C18 H20 F N3 O4 | -47152.3 | 35.2 |
| 84 | Dihydrolycorine | C16 H19 N O4 | -2.68 | 66.1 |
| 85 | Cytisine | C11 H14 N2 O | -400173.81 | 34 |
| 86 | 5-(1,2,4a,5-tetramethyl-7-oxo-3,4,8,8a-tetrahydro-2H-naphthalen-1-yl)-3-methylpentanoic acid | C20 H32 O3 | 68649.8 | 81.4 |
| 87 | Feretoside 6-β-Hydroxygeniposide | C17 H24 O11 | 89013.01 | 54.8 |
| 88 | SPECTINOMYCIN | C14 H24 N2 O7 | -6.27 | 61.8 |
| 89 | NCGC00380949-01_C28H42O5_3,5,9,23-Tetrahydroxy-18,22-cycloergosta-6,8(14)-dien-11-one | C28 H42 O5 | -209515.58 | 30.9 |
| 90 | 1-(m-tolyl)isoquinoline | C16 H13 N | -6.57 | 30 |
| 91 | isomaltulose | C12 H22 O11 | 9.32 | 51.2 |
| 92 | NCGC00180720-03_C20H32O3_1-Naphthalenepentanoic acid, 1,4,4a,5,6,7,8,8a-octahydro-beta,2,5,5,8a-pentamethyl-4-oxo-, (betaS,1S,8aR)- | C20 H32 O3 | 4.15 | 42.9 |
| 93 | GUANINE | C5 H5 N5 O | 12.73 | 32.8 |
| 94 | NCGC00180485-03_C21H32O4_(5beta,8alpha,9beta,10alpha,13alpha)-18-Methoxy-18-oxokauran-17-oic acid | C21 H32 O4 | -63121.46 | 65.5 |
| 95 | L-Glutamic acid | C5 H9 N O4 | 10.38 | 88.2 |
| 96 | 7,8-dimethylalloxazine (lumichrome) | C12 H10 N4 O2 | 6.47 | 45.2 |
| 97 | NCGC00347620-02_C22H34O4_7-Oxabicyclo[4.1.0]hept-3-ene-2,5-diol, 4-(hydroxymethyl)-1-[(1,4,4a,5,6,7,8,8a-octahydro-2,5,5,8a-tetramethyl-1-naphthalenyl)methyl]- | C22 H34 O4 | 4.23 | 38.6 |
| 98 | 14-hydroxy-14-(hydroxymethyl)-5,9-dimethyltetracyclo[11.2.1.0¹,¹⁰.0⁴,⁹]hexadecane-5-carboxylic acid | C20 H32 O4 | 3.009 | 31.2 |
| 99 | GUANOSINE | C10 H13 N5 O5 | 12.37 | 82.2 |
| 100 | RETUSIN 7-METHYL ETHER | C17 H14 O5 | 3.58 | 65.9 |
| 101 | NCGC00380144-01_C20H28O3_(8E)-14-Hydroxy-5,9,12,13-tetramethyl-16-methylene-4-oxatricyclo[10.3.1.0~3,5~]hexadeca-1(15),8-dien-2-one | C20 H28 O3 | 69522.98 | 82.6 |
| 102 | 2-methoxy-5-methyl-3-(2-methylbut-3-en-2-yl)chromen-4-one | C16 H18 O3 | -3.27 | 48.1 |
| 103 | D-GLYCERIC ACID | C3 H6 O4 | 0.73 | 96.4 |
| 104 | NCGC00381393-01_C17H22O4_3,8a-Dimethyl-5-methylene-2-oxo-2,4,4a,5,6,7,8,8a,9,9a-decahydronaphtho[2,3-b]furan-8-yl acetate | C17 H22 O4 | -62070.56 | 32 |
| 105 | Patulin | C7 H6 O4 | 7.34 | 78.1 |
| 106 | Piceatannol | C14 H12 O4 | -2.58 | 53.6 |
| 107 | Epigallocatechin (-) | C15 H14 O7 | 4.22 | 35.6 |
| 108 | NCGC00384756-01_C27H42O4_(3beta,5alpha,8xi,9xi,14xi,25S)-3-Hydroxyspirostan-12-one | C27 H42 O4 | 12.19 | 32.4 |
| 109 | Aconitic Acid | C6 H6 O6 | 8.71 | 85.9 |
| 110 | Loratadine | C22 H23 Cl N2 O2 | -57527.09 | 45.1 |
| 111 | NCGC00169081-02_C23H34O16_beta-D-Glucopyranose, 1-[2-[(2S,3E,4S)-3-ethylidene-2-(beta-D-glucopyranosyloxy)-3,4-dihydro-5-(methoxycarbonyl)-2H-pyran-4-yl]acetate] | C23 H34 O16 | 38826.55 | 43.3 |
| 112 | (-)-Riboflavin | C17 H20 N4 O6 | 58449.96 | 73.7 |
| 113 | Eicosanoids_PGJ2_C20H30O4 | C20 H30 O4 | 65782.98 | 91.7 |
| 114 | Selaginpulvilin S | C35 H26 O5 | -3.31 | 44.3 |
| 115 | 3-[(3S,5S,8R,10S,13R,14S,17R)-3-[4,5-dihydroxy-6-(hydroxymethyl)-3-[3,4,5-trihydroxy-6-(hydroxymethyl)oxan-2-yl]oxyoxan-2-yl]oxy-14-hydroxy-10,13-dimethyl-1,2,3,4,5,6,7,8,9,11,12,15,16,17-tetradecahydrocyclopenta[a]phenanthren-17-yl]-2H-furan-5-one | C35 H54 O14 | 7.18 | 31.4 |
| 116 | NCGC00381111-01_C23H38N4O6_1-[(2E,4E)-6,7-Dihydroxy-2,4-octadienoyl]prolyl-N-methylvalyl-N~2~-methylalaninamide | C23 H38 N4 O6 | -8.76 | 47.3 |
| 117 | NCGC00169582-02_C11H12O4_1H-2-Benzopyran-1-one, 3,4-dihydro-6-hydroxy-8-methoxy-3-methyl- | C11 H12 O4 | 10.88 | 70.8 |
| 118 | 1,4a-dimethyl-9-oxo-7-propan-2-yl-3,4,10,10a-tetrahydro-2H-phenanthrene-1-carboxylic acid | C20 H26 O3 | -3.31 | 44.3 |
| 119 | Putative smenopyrone | C25 H38 O5 | -52549.03 | 76.2 |
| 120 | Nopaline | C11 H20 N4 O6 | -24.96 | 53.4 |
| 121 | Benzomalvin B_120255 | C24 H17 N3 O2 | -2.36 | 31.9 |
| 122 | [(2E,6Z,10Z)-7,11-bis(acetyloxymethyl)-3,15-dimethylhexadeca-2,6,10,14-tetraenyl] acetate | C26 H40 O6 | -49029.42 | 55.4 |
| 123 | kojic acid | C6 H6 O4 | 4.33 | 80.1 |
| 124 | NCGC00380339-01_C15H19NO4_2-Pentenamide, 2-methyl-5-[(1R,3S,6R,9S)-6-methyl-8-oxo-7-oxatricyclo[4.3.0.0~3,9~]non-9-yl]-4-oxo-, (2E)- | C15 H19 N O4 | 79327.02 | 68.2 |
| 125 | Phytosphingosine | C18 H39 N O3 | -100924.94 | 62.6 |
| 126 | Arachidonic_acid | C20 H32 O2 | 4.17 | 30.2 |
| 127 | NCGC00381032-01_C21H32O3_(3R,4R,5R)-3-[(11E)-11,15-Hexadecadien-9-yn-1-yl]-4-hydroxy-5-methyldihydro-2(3H)-furanone | C21 H32 O3 | -96394.24 | 59.7 |
| 128 | L-ARGININE | C6 H14 N4 O2 | 12.23 | 91.3 |
| 129 | NCGC00385238-01_C25H36O4_ | C25 H36 O4 | 5.58 | 70 |
| 130 | 2-methoxy-4-pentadecylbenzoic acid | C23 H38 O3 | 3.95 | 79.1 |
| 131 | PYRIDOXAMINE | C8 H12 N2 O2 | 12.61 | 50.9 |
| 132 | Dictyoceratin A | C23 H32 O4 | -59051.12 | 43.5 |
| 133 | captopril | C9 H15 N O3 S | 82964.64 | 39.5 |
| 134 | MLS002153902-01!Monocrotaline315-22-0 | C16 H23 N O6 | 12.32 | 37.6 |
| 135 | 3-HYDROXYBENZOATE | C7 H6 O3 | 6.28 | 96.6 |
| 136 | Bis(2-ethylhexyl) phthalate | C24 H38 O4 | 4.81 | 77.2 |
| 137 | Captopril (Capoten) | C9 H15 N O3 S | -3.03 | 57.7 |
| 138 | 4-Methylumbelliferone | C10 H8 O3 | 12.08 | 79.5 |
| 139 | Galanthamine hydrobromide | C17 H21 N O3 | -111534.51 | 33.9 |
| 140 | Mefenamic Acid | C15 H15 N O2 | 11.57 | 73 |
| 141 | NCGC00385184-01_C32H36N2O5_chaetoglobin A | C32 H36 N2 O5 | 41613.65 | 80.9 |
| 142 | MLS000028517-01! | C21 H30 O2 | 11.97 | 34.8 |
| 143 | Crocetin | C20 H24 O4 | 4.02 | 47.5 |
| 144 | Gallic acid | C7 H6 O5 | 9 | 91.3 |
| 145 | D-GLUCOSE 6-PHOSPHATE | C6 H13 O9 P | 12.14 | 66.8 |
| 146 | NCGC00385408-01_C17H17N5O2_3-(1H-Imidazol-4-ylmethyl)-6-(1H-indol-3-ylmethyl)-2,5-piperazinedione | C17 H17 N5 O2 | 7.76 | 52.1 |
| 147 | Ligustilide | C12 H14 O2 | -115635.78 | 62.3 |
| 148 | NCGC00385237-01_C30H48O4_ | C30 H48 O4 | -38123.75 | 66.9 |
| 149 | 2-Adamantanone | C10 H14 O | 12.01 | 64.2 |
| 150 | Dipyridamole (Persantine) | C24 H40 N8 O4 | -500002.18 | 44.6 |
| 151 | NCGC00180512-02! | C21 H32 O5 | -499997.55 | 40.1 |
| 152 | Conessine | C24 H40 N2 | 106518.67 | 30.6 |
| 153 | 19,20-epoxynovacine | C24 H28 N2 O6 | 2.8 | 58.6 |
| 154 | DEOXYCARNITINE | C7 H15 N O2 | 11.98 | 38.5 |
| 155 | INDOLE-3-PYRUVIC ACID | C11 H9 N O3 | 13.25 | 49.6 |
| 156 | L-HISTIDINE | C6 H9 N3 O2 | 12.78 | 81.1 |
| 157 | NCGC00169698-03!3-hydroxy-8-methoxy-3-methyl-2,4-dihydrobenzo[a]anthracene-1,7,12-trione | C20 H16 O5 | -53582.66 | 57.2 |
| 158 | Orsellinic acid | C8 H8 O4 | 10.1 | 60.5 |
| 159 | Senecionine | C18 H25 N O5 | -6.76 | 73.1 |
| 160 | Ginkgolic Acid C15:1 | C22 H34 O3 | 4.28 | 51.9 |
| 161 | NCGC00385438-01!1-methoxy-3-methyl-9H-carbazole | C14 H13 N O | -7.47 | 38.1 |
| 162 | Naftifine HCl | C21 H22 Cl N | -111324.27 | 43.9 |
| 163 | Lupinine | C10 H19 N O | 224401.66 | 44.1 |
| 164 | Cryptotanshinone | C19 H20 O3 | 4.19 | 39.2 |
| 165 | NCGC00380781-01_C15H20N2O5_ | C15 H20 N2 O5 | 71345.17 | 69.3 |
| 166 | Adenine | C5 H5 N5 | 12.4 | 83.8 |
| 167 | NCGC00385583-01!3-hydroxy-4-methoxyxanthen-9-one | C14 H10 O4 | 1.7 | 51.7 |
| 168 | NCGC00160208-01!6-Demethoxy-isomaculosidine | C13 H11 N O3 | 11.61 | 30.8 |
| 169 | 3-hydroxy-6-[[(E)-3-hydroxy-2,4-dimethylhept-4-enoyl]amino]-2,4-dimethyl-5-oxohexanoic acid | C17 H29 N O6 | 64052.22 | 42.5 |
| 170 | Mappain | C29 H36 O4 | 6.01 | 54.3 |
| 171 | NCGC00160291-01!Tropan-3-yl-carboxylic acid (2-hydroxy-1-phenyl)-ethyl ester | C17 H23 N O3 | 62296.2 | 37.1 |
| 172 | Isopongaflavone | C21 H18 O4 | 4.77 | 40 |
| 173 | NCGC00384840-01_C17H22O5_9a-Hydroxy-3,8a-dimethyl-5-methylene-2-oxo-2,4,4a,5,6,7,8,8a,9,9a-decahydronaphtho[2,3-b]furan-6-yl acetate | C17 H22 O5 | -58826.87 | 30.7 |
| 174 | Nicotinic acid | C6 H5 N O2 | 4.2 | 79.1 |
| 175 | "2,3-DIHYDROXYBENZOATE" | C7 H6 O4 | 7.24 | 91.2 |
| 176 | 4-ACETOXYPHENOL | C8 H8 O3 | 7.14 | 79.7 |
| 177 | NCGC00386062-01!(3S,6R,12S)-6-benzyl-3-butan-2-yl-9-(6,7-dihydroxyoctyl)-1,4,7,10-tetrazabicyclo[10.4.0]hexadecane-2,5,8,11-tetrone | C31 H48 N4 O6 | -1.04 | 33.3 |
| 178 | NCGC00380526-01_C22H30O6_1-Naphthalenecarboxylic acid, 3-(acetyloxy)-5-[2-(3-furanyl)ethyl]-3,4,4a,5,6,7,8,8a-octahydro-8a-(hydroxymethyl)-5,6-dimethyl- | C22 H30 O6 | -46151.83 | 63.5 |
| 179 | NCGC00381317-01!(E)-1-[2-(hydroxymethyl)pyrrolidin-1-yl]-2-methyldec-8-ene-1,3-dione | C16 H27 N O3 | 3.11 | 47.8 |
| 180 | Sildenafil citrate | C28 H38 N6 O11 S | -644113.24 | 52.9 |
| 181 | NCGC00347544-02!4-hydroxy-3-[(E)-7-hydroxy-3,7-dimethyl-4-oxooct-5-enyl]-5-(3-methylbut-2-enyl)benzoic acid | C22 H30 O5 | 4.19 | 46.2 |
| 182 | (2S)-4-hydroxy-2-(2-hydroxypropan-2-yl)-7-methyl-2,3-dihydrofuro[3,2-g]chromen-5-one | C15 H16 O5 | -3.15 | 38 |
| 183 | NCGC00380552-01_C22H36O3_2(1H)-Naphthalenone, 8-[5-(acetyloxy)-3-methylpentyl]octahydro-4,4,8a-trimethyl-7-methylene- | C22 H36 O3 | -63113.46 | 37.8 |
| 184 | Probenecid (Benemid) | C13 H19 N O4 S | 0.51 | 46.2 |
| 185 | Pentamidine HCl | C19 H26 Cl2 N4 O2 | -174583.35 | 67.1 |
| 186 | NCGC00169194-02_C25H28N4O4_3-[(2-Benzyl-9-hydroxy-3-oxo-2,3,9,9a-tetrahydro-1H-imidazo[1,2-a]indol-9-yl)methyl]-6-isopropyl-2,5-piperazinedione | C25 H28 N4 O4 | -40180.92 | 47.5 |
| 187 | NCGC00384758-01_C27H40O5_(2alpha,3beta,5alpha,8xi,14xi,25S)-2,3-Dihydroxyspirost-9(11)-en-12-one | C27 H40 O5 | 6.04 | 36.7 |
| 188 | dihydroauroglaucin | C19 H24 O3 | 3.85 | 57.9 |
| 189 | Juarezic Acid | C11 H10 O2 | 8.26 | 77.6 |
| 190 | 2-[4a-methyl-8-methylidene-4-(3-methylpentanoyloxy)-1,2,3,4,5,6,7,8a-octahydronaphthalen-2-yl]prop-2-enoic acid | C21 H32 O4 | 3.77 | 48.8 |
| 191 | Ciprofloxacin (Cipro) | C17 H18 F N3 O3 | -7.22 | 59.9 |
| 192 | Pseudostrychnine | C21 H22 N2 O3 | -0.18 | 67.3 |
| 193 | Azatadine maleate | C28 H30 N2 O8 | -444319.42 | 45 |
| 194 | NCGC00385267-01_C15H20O4_2-(6-Hydroxy-3,8-dimethyl-2-oxo-1,2,4,5,6,7,8,8a-octahydro-5-azulenyl)acrylic acid | C15 H20 O4 | -68190.33 | 43.3 |
| 195 | "NCGC00160249-01!2,3-Dihydro-6-Methoxy-4-Methyl-2-Hydroxyethylfuro[3,2-c]quinoline" | C14 H15 N O3 | 11.96 | 42.9 |
| 196 | 5-[(E)-2-(3,5-dihydroxyphenyl)ethenyl]-2-methoxybenzene-1,3-diol | C15 H14 O5 | 10.7 | 44.1 |
| 197 | methyl (1S,12S,13E)-13-ethylidene-18-(hydroxymethyl)-4-methoxy-8,15-diazapentacyclo[10.5.1.0¹,⁹.0²,⁷.0¹⁰,¹⁵]octadeca-2,4,6,8-tetraene-18-carboxylate | C22 H26 N2 O4 | -164861.22 | 64.2 |
| 198 | "1-Naphthaleneheptanoic acid, 1,2,6,7,8,8a-hexahydro-?,?-dihydroxy-2-methyl-8-(2-methyl-1-oxobutoxy)-, methyl ester_120185" | C24 H38 O6 | 52068.88 | 72.2 |
| 199 | Ergosterol Peroxide_120246 | C28 H44 O3 | -51314.39 | 67 |
| 200 | 4-Hydroxybenzoic acid | C7 H6 O3 | 5.35 | 77.7 |
| 201 | NCGC00384901-01_C24H17N3O2_(7Z)-7-Benzylidene-6-methyl-6,7-dihydroquinazolino[3,2-a][1,4]benzodiazepine-5,13-dione | C24 H17 N3 O2 | -1.72 | 37.5 |
| 202 | NCGC00347634-02!2,3-dihydroxy-10,13-dimethyl-2,3,4,5,6,7,8,14,15,17-decahydro-1H-cyclopenta[a]phenanthrene-12,16-dione | C19 H26 O4 | -69081.88 | 39.9 |
| 203 | 13-(acetyloxy)-1-hydroxy-8-(hydroxymethyl)-4,12,12,15-tetramethyl-5-oxotetracyclo[8.5.0.0²,⁶.0¹¹,¹³]pentadeca-3,8-dien-14-yl (2E)-2-methylbut-2-enoate | C27 H36 O7 | 6.93 | 42.4 |
| 204 | Pesticide5_Simetryn_C8H15N5S_Ethanamine, N,N'-[6-(methylthio)-1,3,5-triazine-2,4(1H,3H)-diylidene]bis- | C8 H15 N5 S | -9.93 | 58.9 |
| 205 | Clozapine (Clozaril) | C18 H19 Cl N4 | -499998.41 | 37.1 |
| 206 | Pesticide6_Tebuconazole_C16H22ClN3O_1-(4-Chlorophenyl)-4,4-dimethyl-3-(1H-1,2,4-triazol-1-ylmethyl)-3-pentanol | C16 H22 Cl N3 O | 1.25 | 64.3 |
| 207 | Pesticide2_Thidiazuron_C9H8N4OS_1-Phenyl-3-(1,2,3-thiadiazol-5-yl)urea | C9 H8 N4 O S | -7.77 | 40.1 |
| 208 | phenylethylamide 359 | C24 H41 N O | 5.23 | 44.4 |
| 209 | NCGC00160238-01!RIBALININE | C15 H17 N O3 | -123583.71 | 31.2 |
| 210 | Pesticide9_Pencycuron (Monceren) _C19H21ClN2O_ | C19 H21 Cl N2 O | 2.9 | 42.1 |
| 211 | NCGC00385289-01_C20H24O5_(6beta)-11,12-Dihydroxy-6,20-epoxyabieta-8(14),9(11),12-triene-7,20-dione | C20 H24 O5 | 11.35 | 33.2 |
| 212 | NCGC00385922-01_C15H20O3_(2R,5S,9R,12R)-13,13-Dimethyl-7-oxatetracyclo[7.5.0.0~1,5~.0~2,12~]tetradecane-4,8-dione | C15 H20 O3 | 12.33 | 66.8 |
| 213 | NCGC00380944-01_C30H46O3_(3beta,5xi,9xi,13alpha,17alpha,18xi)-3-Hydroxy-13,28-epoxyurs-11-en-28-one | C30 H46 O3 | 10.18 | 82.3 |
| 214 | Citrazinc acid | C6 H5 N O4 | 7.52 | 59.5 |
| 215 | NCGC00381408-02_C16H24O4_4-Hydroxy-1-methoxy-6,6,9a-trimethyl-4,5,5a,6,7,8,9,9a-octahydronaphtho[1,2-c]furan-3(1H)-one | C16 H24 O4 | -64281.91 | 62.1 |
| 216 | 4-Methoxyphenylacetic acid | C9 H10 O3 | 7.54 | 49.2 |
| 217 | 3-HYDROXYANTHRANILATE | C7 H7 N O3 | 7.53 | 78.1 |
| 218 | Articaine HCl | C13 H21 Cl N2 O3 S | -112392.59 | 53 |
| 219 | 50-22-6 | C21 H30 O4 | 4.21 | 52.4 |
| 220 | Risperidone (Risperdal) | C23 H27 F N4 O2 | 0.01 | 63.2 |
| 221 | Glabrolide | C30 H44 O4 | 6.49 | 73.3 |
| 222 | Tonantzitlolone B | C28 H42 O9 | 42100.1 | 62.4 |
| 223 | Euparin | C13 H12 O3 | 0.57 | 33.4 |
| 224 | (2S,3S)-3,5,7-trihydroxy-6-methyl-2-(3,4,5-trihydroxyphenyl)-2,3-dihydrochromen-4-one | C16 H14 O8 | 4.73 | 48.6 |
| 225 | Cortell | C23 H32 O6 | 5 | 37.7 |
| 226 | 18 β-Glycyrrhetintic Acid | C30 H46 O4 | 4.46 | 75 |
| 227 | Pesticide3_Chloroxuron_C15H15ClN2O2_Urea, N'-[4-(4-chlorophenoxy)phenyl]-N,N-dimethyl- | C15 H15 Cl N2 O2 | 0.84 | 37.8 |
| 228 | Dehydrovariabilin | C17 H14 O4 | -1.38 | 39.3 |
| 229 | NCGC00347650-02_C17H14O6_6H-[1,3]Dioxolo[5,6]benzofuro[3,2-c][1]benzopyran-6a(12aH)-ol, 3-methoxy-, (6aR,12aR)- | C17 H14 O6 | -57339.4 | 34.6 |
| 230 | Piperonylic Acid | C8 H6 O4 | 8.74 | 60.2 |
| 231 | Cinnamic acid | C9 H8 O2 | 6.89 | 51.6 |
| 232 | Codeine | C18 H21 N O3 | -1.21 | 54.1 |
| 233 | Nordazepam | C15 H11 Cl N2 O | 0.21 | 65.7 |
| 234 | NCGC00180346-02_C22H25N5O2_3-(1H-Imidazol-4-ylmethyl)-10b-(2-methyl-3-buten-2-yl)-6,10b,11,11a-tetrahydro-2H-pyrazino[1',2':1,5]pyrrolo[2,3-b]indole-1,4(3H,5aH)-dione | C22 H25 N5 O2 | -81858.15 | 34.7 |
| 235 | β-Asarone | C12 H16 O3 | -105626.61 | 58 |
| 236 | NCGC00381405-01!7-hydroxy-3,5-dimethylisochromen-1-one | C11 H10 O3 | 0.3 | 68.3 |
| 237 | Resibufogenin | C24 H32 O4 | 4.49 | 64.4 |
| 238 | Ketoconazole | C26 H28 Cl2 N4 O4 | -4.66 | 58.5 |
| 239 | Gramine | C11 H14 N2 | 217990.67 | 38.4 |
| 240 | NCGC00015366-03!DUBINIDINE | C15 H17 N O4 | 12.33 | 32.2 |
| 241 | NCGC00386099-01! | C22 H20 O7 | 5.38 | 55.3 |
| 242 | NCGC00386020-01_C18H28O3_8-{(1S,5R)-4-Oxo-5-[(2Z)-2-penten-1-yl]-2-cyclopenten-1-yl}octanoic acid | C18 H28 O3 | -75225.51 | 57.1 |
| 243 | Ergonovine maleate | C19 H23 N3 O2 | -0.26 | 42.5 |
| 244 | NCGC00347666-02!2,4-dihydroxy-6-methyl-3-[(2E,4E)-3-methyl-5-[(1R,2R,6R)-1,2,6-trimethyl-3-oxocyclohexyl]penta-2,4-dienyl]benzaldehyde | C23 H30 O4 | 59374.48 | 37.7 |
| 245 | NCGC00380981-01_C28H40O5_ | C28 H40 O5 | 6.04 | 38.5 |
| 246 | Gardenin B | C19 H18 O7 | 4.32 | 77.1 |
| 247 | Oleic_acid | C18 H34 O2 | 11.14 | 56.9 |
| 248 | "NCGC00160335-01!4,7,7-Trimethyl-2,3-dioxo-bicyclo[2.2.1]heptane-1-carboxylic acid" | C11 H14 O4 | 11.67 | 35 |
| 249 | NCGC00384804-01_C20H30O11_2-(4-Methoxyphenyl)ethyl 6-O-alpha-L-arabinopyranosyl-beta-D-glucopyranoside | C20 H30 O11 | 38159.2 | 31.9 |
| 250 | Phenylalanine | C9 H11 N O2 | 80.95 | 30 |
| 251 | NCGC00381029-01!1-(3-ethyl-2,4-dihydroxy-6-methoxyphenyl)butan-1-one | C13 H18 O4 | -92312.59 | 33.6 |
| 252 | NCGC00180872-02![(2S,3R,4S,5R,6S)-4,5-dihydroxy-6-[5-hydroxy-2-(4-hydroxyphenyl)-4-oxo-7-[(2S,3R,4R,5R,6S)-3,4,5-trihydroxy-6-methyloxan-2-yl]oxychromen-3-yl]oxy-2-methyloxan-3-yl] acetate | C29 H32 O15 | 1.22 | 33 |
| 253 | NCGC00017285-04!HAPLAMINE | C15 H15 N O3 | 11.7 | 41.1 |
| 254 | "2,5-DIHYDROXYBENZOATE" | C7 H6 O4 | 7.34 | 94.5 |
| 255 | Diosgenin | C27 H42 O3 | -53051.68 | 44 |
| 256 | NCGC00380816-01_C11H20O7_ | C11 H20 O7 | 83217.4 | 54.9 |
| 257 | NCGC00385253-01_C30H44O7_(2S,4R,9beta,16alpha,23E)-2,16,20,25-Tetrahydroxy-9,10,14-trimethyl-4,9-cyclo-9,10-secocholesta-5,23-diene-1,11,22-trione | C30 H44 O7 | -34876.46 | 41 |
| 258 | NCGC00380724-01_C28H47NO8_(3R,5R,6S,7S,9R,11E,13S,14R)-14-Ethyl-13-hydroxy-3,5,7,9,13-pentamethyl-2,4,10-trioxooxacyclotetradec-11-en-6-yl 3,4,6-trideoxy-3-(dimethylamino)-D-xylo-hexopyranoside | C28 H47 N O8 | -34276.85 | 41.2 |
| 259 | "(8S,9S,10R,13S,14S,17S)-17-acetyl-10,13-dimethyl-1,2,6,7,8,9,11,12,14,15,16,17-dodecahydrocyclopenta[a]phenanthren-3-one" | C21 H30 O2 | -69952.42 | 70.6 |
| 260 | 4-Methoxysalicylic acid | C8 H8 O4 | 11.21 | 81.4 |
| 261 | verruculotoxin | C15 H20 N2 O | 90038.57 | 45.1 |
| 262 | Riboflavin | C17 H20 N4 O6 | -2084.72 | 30.8 |
| 263 | Shikimic acid | C7 H10 O5 | 9.73 | 66.8 |
| 264 | NCGC00380686-01_C20H26O6_ | C20 H26 O6 | 7.56 | 51.3 |
| 265 | Schweinfurthin F | C30 H38 O5 | 7.16 | 48.9 |
| 266 | methyl 8,11,15,20,22-pentahydroxy-24-methyl-13,18,27-trioxo-6-oxaheptacyclo[15.10.2.0¹,¹⁹.0³,¹⁶.0⁵,¹⁴.0⁷,¹².0²¹,²⁶]nonacosa-3,5(14),11,15,19,21(26),22,24,28-nonaene-7-carboxylate | C31 H24 O11 | 3.5 | 30.9 |
| 267 | "NCGC00160261-01!10b-Methyl-3,4,5,6,10b,11-hexahydro-2H-4b-aza-chrysene-1,12-dione" | C18 H19 N O2 | 78195.98 | 51 |
| 268 | CATECHOL | C6 H6 O2 | 1.73 | 94.9 |
| 269 | Pimobendan (Vetmedin) | C19 H18 N4 O2 | -1.6 | 47.8 |
| 270 | Palmitoyl Ethanolamide | C18 H37 N O2 | 23305.83 | 76.3 |
| 271 | NCGC00380179-01_C30H46O4_(23E)-5,25-Dihydroxy-4,4-dimethylergosta-8,23-diene-3,22-dione | C30 H46 O4 | -106378.54 | 52.7 |
| 272 | NCGC00386074-01_C18H19NO4_ | C18 H19 N O4 | 9.43 | 45.3 |
| 273 | (2R,3R,4S,5S,6R)-2-[(E)-3-(4-hydroxy-3-methoxyphenyl)prop-2-enoxy]-6-(hydroxymethyl)oxane-3,4,5-triol | C16 H22 O8 | 49760.21 | 36.3 |
| 274 | Pesticide6_Triticonazole_C17H20ClN3O_(5E)-5-(4-Chlorobenzylidene)-2,2-dimethyl-1-(1H-1,2,4-triazol-1-ylmethyl)cyclopentanol | C17 H20 Cl N3 O | 1.68 | 33.2 |
| 275 | Linderane | C15 H16 O4 | 2.41 | 54.6 |
| 276 | Picroside II | C23 H28 O13 | 7.17 | 38 |
| 277 | Triamterene | C12 H11 N7 | 2.16 | 59.3 |
| 278 | NCGC00380870-01_C17H14O2_2(5H)-Furanone, 4-phenyl-3-(phenylmethyl)- | C17 H14 O2 | -4.37 | 46.1 |
| 279 | NCGC00160283-01!1-(8-Quinolinyl)-beta-carboline | C20 H13 N3 | -528850.61 | 37.2 |
| 280 | NCGC00386030-01_C15H14O3_3-[(E)-2-(3-Hydroxyphenyl)vinyl]-5-methoxyphenol | C15 H14 O3 | -1.92 | 43.1 |
| 281 | [(2S,3R,4S,5S,6R)-3,4,5-trihydroxy-6-(hydroxymethyl)oxan-2-yl] (2E,6E)-8-hydroxy-2,6-dimethylocta-2,6-dienoate | C16 H26 O8 | 49180.9 | 37.8 |
| 282 | NCGC00380766-01!1,5,8-trihydroxy-3-[(2S,3R,4S,5S,6R)-3,4,5-trihydroxy-6-(hydroxymethyl)oxan-2-yl]oxyxanthen-9-one | C19 H18 O11 | 5.43 | 61.7 |
| 283 | NCGC00169715-02_C20H30O2_Abieta-8(14),9(11),12-triene-7,18-diol | C20 H30 O2 | 72739.68 | 53.5 |
| 284 | teadenol A | C14 H12 O6 | -79622.92 | 32.5 |
| 285 | (7R,8R)-7,8-dihydroxy-3,5,7-trimethyl-8H-isochromen-6-one | C12 H14 O4 | 1.42 | 31.6 |
| 286 | NCGC00180796-02_C28H38O6_(4beta,5beta,6beta,22R)-4,27-Dihydroxy-5,6:22,26-diepoxyergosta-2,24-diene-1,26-dione | C28 H38 O6 | 6.48 | 34.8 |
| 287 | 3-HYDROXYPHENYLACETATE | C8 H8 O3 | 6.74 | 45.9 |
| 288 | lenticin | C14 H18 N2 O2 | -4.34 | 48 |
| 289 | NCGC00168838-03_C30H46O5_(1R,2S,5aR,5bR,7aS,10R,12bR)-2-Hydroxy-10-isopropenyl-3,3,5a,5b,12b-pentamethyloctadecahydrodicyclopenta[a,i]phenanthrene-1,7a(1H)-dicarboxylic acid | C30 H46 O5 | 37040.27 | 34.8 |
| 290 | Ginkgolide B | C20 H24 O10 | 6.05 | 48.5 |
| 291 | Betulinic acid | C30 H48 O3 | 5.6 | 53.6 |
| 292 | methyl 10-acetyloxy-1,2,6a,6b,9,9,12a-heptamethyl-2,3,4,5,6,6a,7,8,8a,10,11,12,13,14b-tetradecahydro-1H-picene-4a-carboxylate | C33 H52 O4 | -152248.19 | 41.6 |
| 293 | 1-[2-hydroxy-4-(3-hydroxy-5-methylphenoxy)-6-methylphenyl]-3-methylbutane-2,3-diol | C19 H24 O5 | 51252.82 | 31.1 |
| 294 | Bisdemethoxycurcumin | C19 H16 O4 | 3.67 | 31.2 |
| 295 | HYDROQUINIDINE | C20 H26 N2 O2 | -9.86 | 82.2 |
| 296 | (3aS,5R,5aS,8aS,9aR)-5,8a-dimethyl-1-methylidene-3a,4,5,5a,6,7,9,9a-octahydroazuleno[6,7-b]furan-2,8-dione | C15 H20 O3 | -19967.64 | 62.9 |
| 297 | NCGC00169592-02_C29H46O4_3aH-Cyclopenta[a]chrysene-3a-carboxylic acid, 1-acetyleicosahydro-9-hydroxy-5a,5b,8,8,11a-pentamethyl-, (1R,3aS,5aR,5bR,9S,11aR)- | C29 H46 O4 | -39289.04 | 46.6 |
| 298 | Pesticide6_Paclobutrazol_C30H40Cl2N6O2_1H-1,2,4-Triazole-1-ethanol, beta-[(4-chlorophenyl)methyl]-alpha-(1,1-dimethylethyl)- | C15 H20 Cl N3 O | 1.09 | 40.9 |
| 299 | Methyl Fexofenadine | C33 H41 N O4 | 2.76 | 45 |
| 300 | NCGC00187466-03!(E)-1-(5-hydroxy-2,2-dimethylchromen-6-yl)-3-phenylprop-2-en-1-one | C20 H18 O3 | -134021.49 | 46.2 |
| 301 | Ursolic acid | C30 H48 O3 | -39454.37 | 65 |
| 302 | Corylin | C20 H16 O4 | -68667.24 | 67.8 |
| 303 | "3,4-DIHYDROXY-L-PHENYLALANINE" | C9 H11 N O4 | -208171.28 | 39.5 |
| 304 | ADENINE | C5 H5 N5 | 5.59 | 78.3 |
| 305 | 15-Deoxy-goyazensolide | C19 H20 O6 | 63846.64 | 87.7 |
| 306 | NCGC00385760-01!(2S,3S,9R,13R,14S,16R)-2,3,16-trihydroxy-17-[(2R)-2-hydroxy-6-methyl-3-oxohept-6-en-2-yl]-4,4,9,13,14-pentamethyl-1,2,3,7,8,10,12,15,16,17-decahydrocyclopenta[a]phenanthren-11-one | C30 H46 O6 | 5.98 | 33.1 |
| 307 | NCGC00169449-02_C15H22O8_beta-D-Glucopyranoside, 4-(2-hydroxyethyl)-2-methoxyphenyl | C15 H22 O8 | 51569.29 | 35.4 |
| 308 | Cotoin | C14 H12 O4 | -3.09 | 49.1 |
| 309 | NCGC00180705-02!(E)-5-hydroxy-1,7-diphenylhept-1-en-3-one | C19 H20 O2 | 78473.59 | 32.6 |
| 310 | NCGC00380243-01! | C22 H20 O8 | 8.47 | 45.2 |
| 311 | 3,4-DIMETHOXYDALBERGIONE | C17 H16 O4 | -0.98 | 32.6 |
| 312 | Orotic Acid | C5 H4 N2 O4 | 6.92 | 78.6 |
| 313 | Imiquimod | C14 H16 N4 | 6.33 | 35.6 |
| 314 | Selaginpulvilin A | C34 H24 O5 | -4.25 | 51.4 |
| 315 | altenusin | C15 H14 O6 | 9.22 | 47.4 |
| 316 | Caffeic acid | C9 H8 O4 | 10.71 | 34.9 |
| 317 | NCGC00385977-01_C16H24O5_4-Hydroxy-5-methoxy-4-[2-methyl-3-(3-methyl-2-buten-1-yl)-2-oxiranyl]-1-oxaspiro[2.5]octan-6-one | C16 H24 O5 | 74229.62 | 58.8 |
| 318 | 2,4,5-trimethoxybenzoic acid | C10 H12 O5 | 11.62 | 37.9 |
| 319 | [(E)-3-(4-methoxyphenyl)prop-2-enyl] (Z)-2-[[(E)-2-methylbut-2-enoyl]oxymethyl]but-2-enoate | C20 H24 O5 | 3.98 | 35.4 |
| 320 | D-(-)-salicin | C13 H18 O7 | 59503.13 | 46.1 |
| 321 | Terpestacin_130136 | C25 H38 O4 | 5.42 | 45.5 |
| 322 | PYROCATECHUIC ACID | C7 H6 O4 | 7.34 | 83.4 |
| 323 | Pinosylvin | C14 H12 O2 | -6.2 | 52.7 |
| 324 | Strychnine | C21 H22 N2 O2 | -0.88 | 65.1 |
| 325 | NCGC00384736-01_C30H44O5_(24E)-3-Oxolanosta-8,24-diene-21,26-dioic acid | C30 H44 O5 | -37180.06 | 34.7 |
| 326 | NCGC00386010-01_C24H36O15_Benzyl beta-D-glucopyranosyl-(1->2)-[beta-D-xylopyranosyl-(1->6)]-beta-D-glucopyranoside | C24 H36 O15 | -5.39 | 33.3 |
| 327 | Propylparaben | C10 H12 O3 | 9.22 | 46.1 |
| 328 | DL-Palmitoylcarnitine | C23 H45 N O4 | 49.06 | 59.6 |
| 329 | NCGC00384878-01_C17H26O10_2H-Pyran-5-carboxylic acid, 3-ethenyl-2-(beta-D-glucopyranosyloxy)-3,4-dihydro-4-(2-hydroxyethyl)-, methyl ester, (2S,3R,4S)- | C17 H26 O10 | 56338.7 | 39.2 |
| 330 | [3,4,5-trihydroxy-6-[4-hydroxy-2-[(1,2,3,6-tetrahydroxycyclohexanecarbonyl)oxymethyl]phenoxy]oxan-2-yl]methyl benzoate | C27 H32 O14 | 37898.45 | 50.5 |
| 331 | Fluvoxamine maleate | C19 H25 F3 N2 O6 | -267198.76 | 53.2 |
| 332 | N-ACETYL-DL-METHIONINE | C7 H13 N O3 S | -3.9 | 33.7 |
| 333 | Pesticide6_Cyproconazole Isomer 1*_C15H18ClN3O_ | C15 H18 Cl N3 O | 61869.46 | 69.4 |
| 334 | TOBRAMYCIN | C18 H37 N5 O9 | 36438.25 | 34.2 |
| 335 | NCGC00180523-02!4,8-dihydroxy-7-(hydroxymethyl)-6-methoxy-3,4-dihydro-2H-naphthalen-1-one | C12 H14 O5 | -75647.69 | 37.2 |
| 336 | NCGC00180856-03!5,6,8-trihydroxy-2-methyl-9-(5,6,8-trihydroxy-2-methyl-4-oxo-2,3-dihydrobenzo[g]chromen-9-yl)-2,3-dihydrobenzo[g]chromen-4-one | C28 H22 O10 | 6.89 | 53.2 |
| 337 | 5,7-O-Diacetyl-14-O-benzoyl-3-O-propanoylmyrsinol | C34 H42 O10 | 36022.59 | 47.1 |
| 338 | Wieland-Gumlich aldehyde | C19 H22 N2 O2 | -1.54 | 40.6 |
| 339 | NCGC00385107-01_C23H32O9_Cyclohexaneacetic acid, 6-[4-(acetyloxy)-3-hydroxy-2-methylene-1-oxobutoxy]-4-ethenyl-2-hydroxy-3-[1-(hydroxymethyl)ethenyl]-4-methyl-alpha-methylene-, methyl ester | C23 H32 O9 | -39823.49 | 34.5 |
| 340 | Fraxinellone | C14 H16 O3 | -94704.14 | 72.3 |
| 341 | Pogostone | C12 H16 O4 | 0.62 | 60.4 |
| 342 | conocarpan | C18 H18 O2 | 63976.24 | 36.4 |
| 343 | Gitogenin | C27 H44 O4 | 1.73 | 46.1 |
| 344 | NCGC00347842-02_C21H34O4_1-Naphthalenepentanoic acid, decahydro-5-(methoxycarbonyl)-beta,5,8a-trimethyl-2-methylene- | C21 H34 O4 | 4.72 | 34.1 |
| 345 | Sodium Tetradecyl Sulfate | C14 H29 Na O4 S | 1.22 | 32.5 |
| 346 | Alternariol monomethyl ether_120111 | C15 H12 O5 | 11.18 | 51.8 |
| 347 | Gentisic acid | C7 H6 O4 | 6.74 | 79.4 |
| 348 | Palmatine | C21 H21 N O4 | 0.59 | 57.2 |
| 349 | NCGC00017285-06_C15H15NO3_9-Methoxy-2,2-dimethyl-2,6-dihydro-5H-pyrano[3,2-c]quinolin-5-one | C15 H15 N O3 | -159573.4 | 31.2 |
| 350 | Vanillin | C8 H8 O3 | 8.6 | 78.7 |
| 351 | NCGC00186632-02_C21H22N2O2_Strychnidin-10-one | C21 H22 N2 O2 | -122772.65 | 65 |
| 352 | Triptonide | C20 H22 O6 | 11.34 | 56.2 |
| 353 | Sunitinib Malate (Sutent) | C26 H33 F N4 O7 | -251810.51 | 46 |
| 354 | Ergosterol Acetate | C32 H50 O2 | 6.76 | 50.2 |
| 355 | NCGC00381050-01!7-(5,7-dimethoxy-4-oxochromen-2-yl)-6-hydroxyheptanoic acid | C18 H22 O7 | 4.36 | 57.1 |
| 356 | 3-phenyllactic acid | C9 H10 O3 | 7.41 | 81.2 |
| 357 | Ferulic acid | C10 H10 O4 | 10.2 | 52.7 |
| 358 | Sudan III | C22 H16 N4 O | -1.24 | 62 |
| 359 | Arenobufagin | C24 H32 O6 | 2.43 | 35.4 |
| 360 | Mundulone Acetate | C28 H28 O7 | 79701.61 | 43.7 |
| 361 | (1R,3R,4R,4aS,5'S,8aR)-5'-(furan-3-yl)-1-hydroxy-8,8a-bis(hydroxymethyl)-3-methylspiro[1,2,3,4a,5,6-hexahydronaphthalene-4,3'-oxolane]-2'-one | C20 H26 O6 | 47007.64 | 30.3 |
| 362 | Goniomedine A | C40 H49 N4 O4 | -35397.92 | 34.7 |
| 363 | Rheochrysidin_120098 | C16 H12 O5 | 14.93 | 81.1 |
| 364 | Hirsuteine | C22 H26 N2 O3 | -1.55 | 68.9 |
| 365 | PHENYLACETIC ACID | C8 H8 O2 | 4.36 | 63 |
| 366 | NCGC00180745-02_C28H36O5_2-Picenecarboxylic acid, 1,2,3,4,4a,5,6,6a,8,12b,13,14,14a,14b-tetradecahydro-10,11-dihydroxy-2,4a,6a,12b,14a-pentamethyl-8-oxo-, (2R,4aS,6aS,12bR,14aS,14bR)- | C28 H36 O5 | 6.7 | 42 |
| 367 | NCGC00380084-01_C21H30O4_Benzo[b]cyclopenta[e]pyran-8(3H)-one, 3a,5,6,7,9,9a-hexahydro-7-hydroxy-1-[(4E)-6-hydroxy-1,5-dimethyl-4-hexen-1-yl]-3a-methyl- | C21 H30 O4 | 3.51 | 32.5 |
| 368 | (2Z)-2-benzylidene-6-methoxy-1-benzofuran-3(2H)-one | C16 H12 O3 | 0.67 | 41.3 |
| 369 | NCGC00169052-03_C23H26O5_6H-Furo[2,3-h]-2-benzopyran-6,8(6aH)-dione, 9-acetyl-3-[(1E,3E,5S)-3,5-dimethyl-1,3-heptadien-1-yl]-9,9a-dihydro-6a-methyl-, (6aR,9R,9aR)- | C23 H26 O5 | 3.18 | 32.5 |
| 370 | Schweinfurthin E | C30 H38 O6 | -44466.65 | 65.1 |
| 371 | Retrochalcone | C16 H14 O4 | -540691.81 | 49.5 |
| 372 | 4-AMINOBENZOATE | C7 H7 N O2 | 6.15 | 52.2 |
| 373 | Cholic Acid | C24 H40 O5 | 4.72 | 33.1 |
| 374 | suberosin | C15 H16 O3 | 69742.18 | 30.1 |
| 375 | Carpachromene | C20 H16 O5 | 4.28 | 34.5 |
| 376 | Sulindac (Clinoril) | C20 H17 F O3 S | 9.26 | 35 |
| 377 | NCGC00380974-01!4,6-dihydroxy-3-methyl-2,3-dihydroisoindol-1-one | C9 H9 N O3 | -0.28 | 37.4 |
| 378 | NCGC00347586-02_C30H46O6_3,19-Dihydroxyurs-12-ene-23,28-dioic acid | C30 H46 O6 | -191148.66 | 37.3 |
| 379 | NCGC00385544-01_C21H26O6_(2S,3R,3aS,7aR)-5-Allyl-2-(4-hydroxy-3-methoxyphenyl)-3a,7a-dimethoxy-3-methyl-3,3a,7,7a-tetrahydro-1-benzofuran-6(2H)-one | C21 H26 O6 | -48129.77 | 31.7 |
| 380 | NCGC00169399-03!(2R,6R)-6-[(3R,10S,12S,13R,17R)-3-(2-carboxyacetyl)oxy-12-hydroxy-4,4,10,13,14-pentamethyl-2,3,5,6,7,11,12,15,16,17-decahydro-1H-cyclopenta[a]phenanthren-17-yl]-2-methyl-3-methylideneheptanoic acid | C34 H52 O7 | -69865.45 | 39.2 |
| 381 | NCGC00380217-01_C20H28O4_4a(2H)-Phenanthrenecarboxylic acid, 7-ethenyl-1,3,4,4b,5,6,7,9,10,10a-decahydro-4b-hydroxy-1,1,7-trimethyl-9-oxo- | C20 H28 O4 | -54212.42 | 40.9 |
| 382 | _130032 | C18 H27 N O5 | 14703.86 | 49 |
| 383 | Leupeptin analogue LVR (or VLR) | C19 H36 N6 O4 | 11.32 | 56.2 |
| 384 | (5S)-5-hydroxy-1,7-diphenylheptan-3-one | C19 H22 O2 | -35591.08 | 57.8 |
| 385 | 4-METHYLCATECHOL | C7 H8 O2 | 145190.01 | 82.3 |
| 386 | Vedelianin | C29 H36 O6 | 7.85 | 62.9 |
| 387 | Dihydrosphingosine | C18 H39 N O2 | 11.86 | 37.8 |
| 388 | 3-hydroxy-2-[[1-[2-[(4-hydroxybenzoyl)amino]-3-methylbutanoyl]pyrrolidine-2-carbonyl]amino]benzoic acid | C24 H27 N3 O7 | 34047.48 | 43.3 |
| 389 | XANTHINE | C5 H4 N4 O2 | 7.36 | 31.6 |
| 390 | Gestodene | C21 H26 O2 | -1.66 | 57.8 |
| 391 | Mycophenolic acid methyl ester_130118 | C18 H22 O6 | 65798.73 | 63.7 |
| 392 | PAEDEROSIDE | C18 H22 O11 S | -49279.38 | 30.5 |
| 393 | Ginkgolide C | C20 H24 O11 | 10.76 | 39.9 |
| 394 | Pyrimethamine | C12 H13 Cl N4 | -3.69 | 52.1 |
| 395 | "2,5-Piperazinedione, 3-[[1-(1,1-dimethyl-2-propen-1-yl)-1H-indol-3-yl]methyl]-6-[[2-(1,1-dimethyl-2-propen-1-yl)-1H-indol-3-yl]methyl]-, (3S,6S)_120157" | C32 H36 N4 O2 | 4.75 | 32.5 |
| 396 | NCGC00169685-02_C29H44O8_Stigmast-7-ene-6,26-dione, 26,28-epoxy-2,3,14,20,22-pentahydroxy-, (2beta,3beta,5beta,9xi,22R,24S,25S,28R)- | C29 H44 O8 | -34612.16 | 31.4 |
| 397 | KOBUSONE | C14 H22 O2 | 170855.68 | 69.8 |
| 398 | (1'S,3R,3'R,5R,6'R,7'S)-5-(furan-3-yl)-3',6',7'-trimethyl-9'-oxaspiro[oxolane-3,2'-tricyclo[5.2.2.0¹,⁶]undecane]-2,8'-dione | C20 H24 O5 | 63877.99 | 49.7 |
| 399 | Atractylodin | C13 H10 O | -93524 | 31.3 |
| 400 | cyclo(Trp-Ala) | C14 H15 N3 O2 | -159553.82 | 68.4 |
| 401 | NCGC00384527-01!2-(3,4-dihydroxyphenyl)-5,8-dihydroxy-7-methoxy-3-[(2S,3R,4R,5R,6S)-3,4,5-trihydroxy-6-methyloxan-2-yl]oxychromen-4-one | C22 H22 O12 | 96227.2 | 36 |
| 402 | NCGC00169067-03! | C21 H20 O8 | 6.39 | 31.3 |
| 403 | Prim-O-glucosylcimifugin | C22 H28 O11 | 7.05 | 66.8 |
| 404 | 20(S)-Protopanaxadiol | C30 H52 O3 | 47759.23 | 45.1 |
| 405 | Pesticide3_Fluometuron_C10H11F3N2O_Urea, N,N-dimethyl-N'-[3-(trifluoromethyl)phenyl]- | C10 H11 F3 N2 O | -10.05 | 38.3 |
| 406 | TRIPTOLIDE | C20 H24 O6 | 10.98 | 51.6 |
| 407 | 3-(4-Hydroxyphenyl)-1-propanol | C9 H12 O2 | 7.38 | 44.2 |
| 408 | NCGC00180676-03_C20H36O3_1-Naphthalenepentanoic acid, decahydro-2-hydroxy-beta,2,5,5,8a-pentamethyl-, (betaS,1R,2R,8aS)- | C20 H36 O3 | 67801.37 | 49.6 |
| 409 | Heteropeucenin, Methyl Ether | C16 H18 O4 | 3.64 | 48.2 |
| 410 | NCGC00169490-02!3-butan-2-yl-4-methyl-2,3-dihydropyrrolo[3,4-b]quinoline-1,9-dione | C16 H18 N2 O2 | -3.04 | 33.2 |
| 411 | Tetrahydroxyxanthone | C13 H8 O6 | -84533.08 | 73.3 |
| 412 | 3-(5,7-dimethoxy-4-oxochromen-2-yl)propanoic acid | C14 H14 O6 | -115166.9 | 45.4 |
| 413 | 3-[(E)-2-(3-hydroxyphenyl)ethenyl]-5-methoxyphenol | C15 H14 O3 | -0.79 | 36.3 |
| 414 | Mandelic Acid, Methyl Ester | C9 H10 O3 | 8.96 | 76.1 |
| 415 | 2',6'-dihydroxy-4'-methoxydihydrochalcone | C16 H16 O4 | 5.08 | 30.7 |
| 416 | NCGC00381425-01!8-hydroxy-8-(3-octyloxiran-2-yl)octanoic acid | C18 H34 O4 | 10.8 | 47 |
| 417 | NCGC00385948-01_C16H17NO4_6,11-Methano-6H-benzo[b]-1,3-benzodioxolo[5,6-e]azepine-8,9-diol, 5,6a,7,8,9,11-hexahydro- | C16 H17 N O4 | -76560.41 | 47.8 |
| 418 | NCGC00385192-01_C29H36O6_(2E,4aR,6R,7S,8E,10R)-10-Acetoxy-4a-hydroxy-1,1,3,6,9-pentamethyl-4-oxo-1a,4,4a,5,6,7,7a,10,11,11a-decahydro-1H-cyclopenta[a]cyclopropa[f][11]annulen-7-yl benzoate | C29 H36 O6 | 6.9 | 32.3 |
| 419 | 3,4-Dihydroxyphenylethanol | C8 H10 O3 | 7.48 | 30.2 |
| 420 | NCGC00168976-02!5-methoxy-2,2-dimethylpyrano[3,2-g]chromen-8-one | C15 H14 O4 | 2.97 | 34.4 |
| 421 | "NCGC00160205-01!N-methyl-2,4-Dihydroxy-3-phenylquinoline" | C16 H13 N O2 | -6.33 | 51.4 |
| 422 | 2-methyl-2-(3-pentadecylphenoxy)propanoic acid | C25 H42 O3 | 56329.09 | 69.3 |
| 423 | Securinine | C13 H15 N O2 | -147519.75 | 68.6 |
| 424 | Harmol Hydrochloride | C12 H10 N2 O | 277577.76 | 38.9 |
| 425 | Tanshinone IIA | C19 H18 O3 | 7.38 | 49.6 |
| 426 | Demethoxyyangonin | C14 H12 O3 | -6.21 | 58.7 |
| 427 | dehydrocurvularin | C16 H18 O5 | 1.8 | 57.2 |
| 428 | NCGC00380535-01_C20H28O3_12-Oxopimara-9(11),15-dien-18-oic acid | C20 H28 O3 | 202460.58 | 35.4 |
| 429 | Bufotalin | C26 H36 O6 | 6.11 | 46.4 |
| 430 | NCGC00384589-01![(2R,3R,4S,5R,6R)-6-[2-(3,4-dihydroxyphenyl)ethoxy]-3,5-dihydroxy-4-[(2S,3R,4R,5R,6S)-3,4,5-trihydroxy-6-methyloxan-2-yl]oxyoxan-2-yl]methyl (E)-3-(4-hydroxy-3-methoxyphenyl)prop-2-enoate | C30 H38 O15 | -9.07 | 46.8 |
| 431 | 4-({5-[5-hydroxy-3-({[(2Z)-2-methylbut-2-enoyl]oxy}methyl)pentyl]-8a-(hydroxymethyl)-5,6-dimethyl-3,4,4a,5,6,7,8,8a-octahydronaphthalen-1-yl}methoxy)-4-oxobutanoic acid | C29 H46 O8 | 42093.15 | 52.1 |
| 432 | NCGC00347775-02!(Z)-5-[(1S,2R,4aR,8aR)-5-(hydroxymethyl)-1,2,4a-trimethyl-2,3,4,7,8,8a-hexahydronaphthalen-1-yl]-3-(hydroxymethyl)pent-2-enoic acid | C20 H32 O4 | 136836.49 | 38.9 |
| 433 | NCGC00169741-02!2-(3,4-dihydroxyphenyl)-5-hydroxy-3,6,7-trimethoxychromen-4-one | C18 H16 O8 | 9.59 | 33.2 |
| 434 | 3-Furfuryl 2-pyrrolecarboxylate | C10 H9 N O3 | -1.03 | 30.2 |
| 435 | NCGC00380444-01_C16H20O3_2-Naphthaleneacetic acid, 1,2,3,7,8,8a-hexahydro-8,8a-dimethyl-alpha-methylene-7-oxo-, methyl ester, (2R,8R,8aR)- | C16 H20 O3 | -69238.02 | 31.5 |
| 436 | NCGC00381330-01_C18H20N2O9_2,4(1H,3H)-Pyrimidinedione, 1-[3-O-(4-hydroxy-2-methoxy-6-methylbenzoyl)-alpha-D-ribofuranosyl]- | C18 H20 N2 O9 | -4.97 | 30.9 |
| 437 | 4-[2-[(1R,4aS,5R,8aS)-6-hydroxy-5-(hydroxymethyl)-5,8a-dimethyl-2-methylidene-3,4,4a,6,7,8-hexahydro-1H-naphthalen-1-yl]ethyl]-2H-furan-5-one | C20 H30 O4 | 7.37 | 36.8 |
| 438 | Noreugenin | C10 H8 O4 | 10.52 | 75.8 |
| 439 | 4-methylumbelliferone | C10 H8 O3 | 8.77 | 61.7 |
| 440 | Vicine | C10 H16 N4 O7 | -8.79 | 51.8 |
| 441 | (2E,4E)-N-(2-methylpropyl)-6-thiophen-2-ylhexa-2,4-dienamide | C14 H19 N O S | -96342.15 | 34.3 |
| 442 | NCGC00385486-01_C25H34O5_2-Butenoic acid, 2-methyl-, (2R,3R,5R)-2,6,6,9-tetramethyl-11-oxotricyclo[5.4.0.0~2,8~]undec-9-ene-3,5-diyl ester, (2Z,2'Z)- | C25 H34 O5 | 96541.96 | 55.9 |
| 443 | methyl (1R,18R)-17-ethyl-3,13-diazapentacyclo[13.3.1.0²,¹⁰.0⁴,⁹.0¹³,¹⁸]nonadeca-2(10),4(9),5,7,16-pentaene-1-carboxylate | C21 H24 N2 O2 | -136846.94 | 32.4 |
| 444 | NCGC00347563-02![1,3-dihydroxy-1-(7-methoxy-2-oxochromen-6-yl)-3-methylbutan-2-yl] (E)-2-methylbut-2-enoate | C20 H24 O7 | -3.24 | 48 |
| 445 | [(2E)-2-hexa-2,4-diynylidene-1,6-dioxaspiro[4.4]nona-3,7-dien-9-yl] 3-methylbut-2-enoate | C18 H16 O4 | 999986.91 | 36.7 |
| 446 | Acitretin | C21 H26 O3 | -0.57 | 34 |
| 447 | reynosin | C15 H20 O3 | -12368.62 | 32.2 |
| 448 | Carnosine | C9 H14 N4 O3 | 167874.69 | 32.2 |
| 449 | NCGC00380822-01!1-[(Z)-but-2-en-2-yl]-8-chloro-3,9-dihydroxy-4,7-dimethylbenzo[b][1,4]benzodioxepin-6-one | C19 H17 Cl O5 | -5.07 | 34.5 |
| 450 | [(2S,3S)-2-[(E,3R,4S)-3,4-dihydroxypent-1-enyl]-6-oxo-2,3-dihydropyran-3-yl] (E)-2-methylbut-2-enoate | C15 H20 O6 | 53942.08 | 35.8 |
| 451 | Citric acid | C6 H8 O7 | 89.26 | 91.7 |
| 452 | Telmisartan (Micardis) | C33 H30 N4 O2 | -521376.86 | 35.8 |
| 453 | Dihydrotanshinone I | C18 H14 O3 | -136481.87 | 41.9 |
| 454 | Linagliptin (BI-1356) | C25 H28 N8 O2 | 1.33 | 62.2 |
| 455 | NCGC00180731-02_C19H26O12_Methyl 2-{[6-O-(beta-D-xylopyranosyl)-beta-D-glucopyranosyl]oxy}benzoate | C19 H26 O12 | 38160.45 | 32.6 |
| 456 | NCGC00169629-02_C27H28N2O4_Benzenepropanamide, N-[2-(acetyloxy)-1-(phenylmethyl)ethyl]-alpha-(benzoylamino)- | C27 H28 N2 O4 | -72104.42 | 60.6 |
| 457 | Iloperidone (Fanapt) | C24 H27 F N2 O4 | -8.9 | 50.2 |
| 458 | Hapalindole D | C21 H24 N2 S | 11.57 | 48.3 |
| 459 | (1R,3S,5S)-8-methyl-8-azabicyclo[3.2.1]octan-3-yl 3-hydroxy-2-phenylpropanoate | C17 H23 N O3 | 131265.67 | 57.4 |
| 460 | Ampicillin | C16 H19 N3 O4 S | -51.98 | 32.8 |
| 461 | 2-[[3,4-dihydroxy-4-(hydroxymethyl)oxolan-2-yl]oxymethyl]-6-[(6,6-dimethyl-4-bicyclo[3.1.1]hept-3-enyl)methoxy]oxane-3,4,5-triol | C21 H34 O10 | -22511.34 | 34.7 |
| 462 | Pesticide4_Halofenozide_C18H19ClN2O2_N'-Benzoyl-4-chloro-N'-(2-methyl-2-propanyl)benzohydrazide | C18 H19 Cl N2 O2 | 54564.35 | 35.9 |
| 463 | butyrolactone i | C24 H24 O7 | 42469.63 | 30.7 |
| 464 | 21-hydroxy-10-methoxy-1,4,14,19,19-pentamethyl-2,7,18-trioxapentacyclo[11.9.0.0³,¹¹.0⁵,⁹.0¹⁴,²⁰]docosa-3(11),4,9-triene-8,17-dione | C25 H32 O7 | 8.72 | 32.9 |
| 465 | 13-OxoODE | C18 H30 O3 | 74681.29 | 62.1 |
| 466 | Pesticide3_Neburon_C12H16Cl2N2O_Urea, N-butyl-N'-(3,4-dichlorophenyl)-N-methyl- | C12 H16 Cl2 N2 O | -80210.53 | 59 |
| 467 | Enalaprilat dihydrate | C18 H28 N2 O7 | -93757.74 | 54.5 |
| 468 | NCGC00168943-02![(2R,3S,4S,5R,6S)-6-[2-(3,4-dihydroxyphenyl)-5,7-dihydroxy-4-oxochromen-3-yl]oxy-3,4,5-trihydroxyoxan-2-yl]methyl acetate | C23 H22 O13 | 3.36 | 41 |
| 469 | Agarotetrol | C17 H18 O6 | 9.46 | 32.8 |
| 470 | 4-methylumbelliferyl acetate | C12 H10 O4 | 5.84 | 59 |
| 471 | 8-hydroxy-2,7,7,11,15-pentamethyl-5,12,16-trioxapentacyclo[9.8.0.0²,⁸.0⁴,⁶.0¹³,¹⁸]nonadec-13(18)-ene-3,17-dione | C21 H28 O6 | 47878.16 | 44.2 |
| 472 | Pterostilbene | C16 H16 O3 | -179634.06 | 49.8 |
| 473 | Jatrorrhizine | C20 H19 N O4 | -560847.35 | 67.5 |
